# Supplementary material for: Cerebellar stimulation prevents Levodopa-induced dyskinesia in mice and normalizes activity in a motor network
Source: Nat Commun. 2022 Jun 9;13:3211. doi: 10.1038/s41467-022-30844-0 (PMC9184492; doi:10.1038/s41467-022-30844-0)
Supplement: Supplementary file 1 — Supplementary Information [file 41467_2022_30844_MOESM1_ESM.pdf]

Supplementary Figure S1

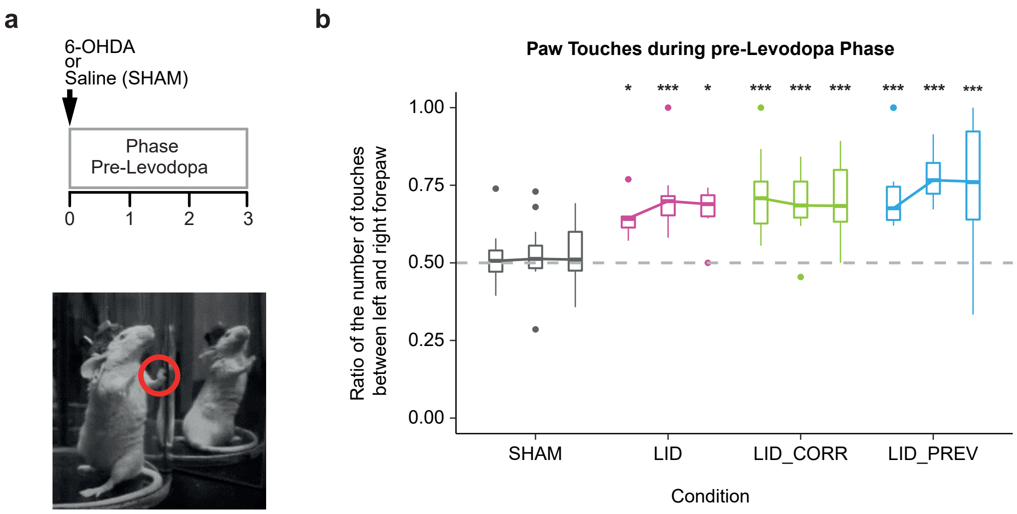

Supplementary Fig. S1 Dopaminergically depleted mice present parkinsonian-like symptoms in the pre-Levodopa phase. Related to Fig. 1

a *Upper*: Experimental timeline of the pre-Levodopa phase. *Bottom*: Image representing a mouse touching the wall of a cylinder (red circle) with its forepaw during test of the asymmetrical use of the forepaw, also called cylinder test. **b** Boxplot showing the averaged ratio of the number of touches between the left (ipsilateral-to-the-lesion) and the right (contralateral-to-the-lesion) forepaws measured over 3 weeks, prior to levodopa treatment, in SHAM (grey, N=15), LID (magenta, N=7), LID_CORR (green, N=17), and LID_PREV (blue, N=8). Boxplots represent the lower and the upper quartiles and horizontal bars in boxplots represent median score. Vertical lines represent the median +/- standard deviation. Isolated points represent outliers of the distribution. Dashed grey line represents the 0.5 ratio corresponding to a symmetrical use of the two forepaws. Welch Anova with two-sided Games Howell post-hoc test and one-way Anova’s with two-sided Tukey post-hoc test based on Levene test. ***$p < 0.001$; **$p < 0.01$; *$p < 0.05$; ns: $p > 0.5$. Source data are provided as a Source Data file. See also Table S2.

Supplementary Figure S2

a

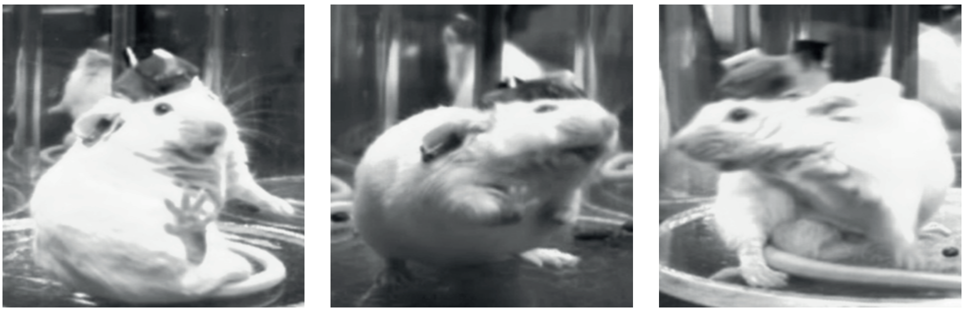

b

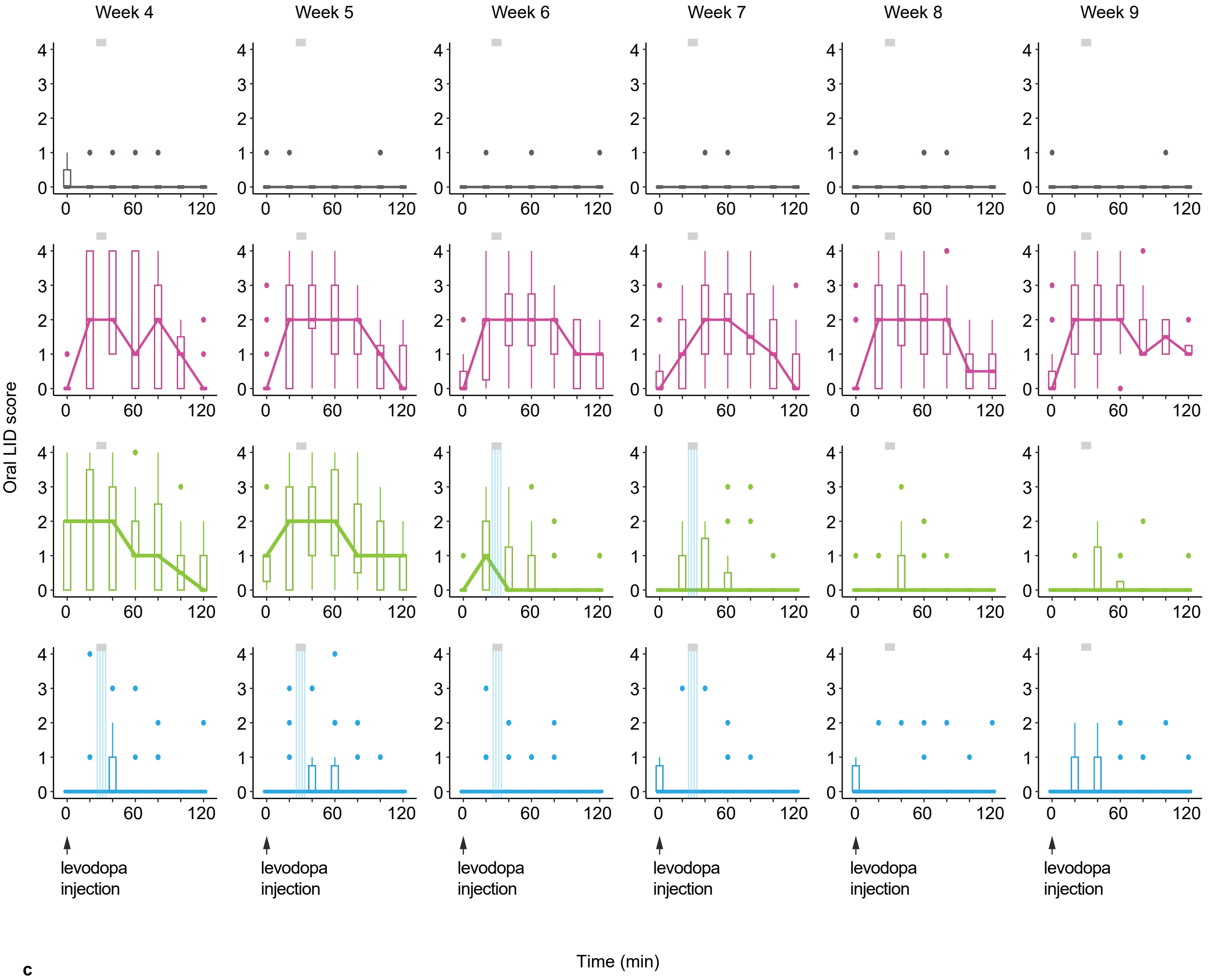

c

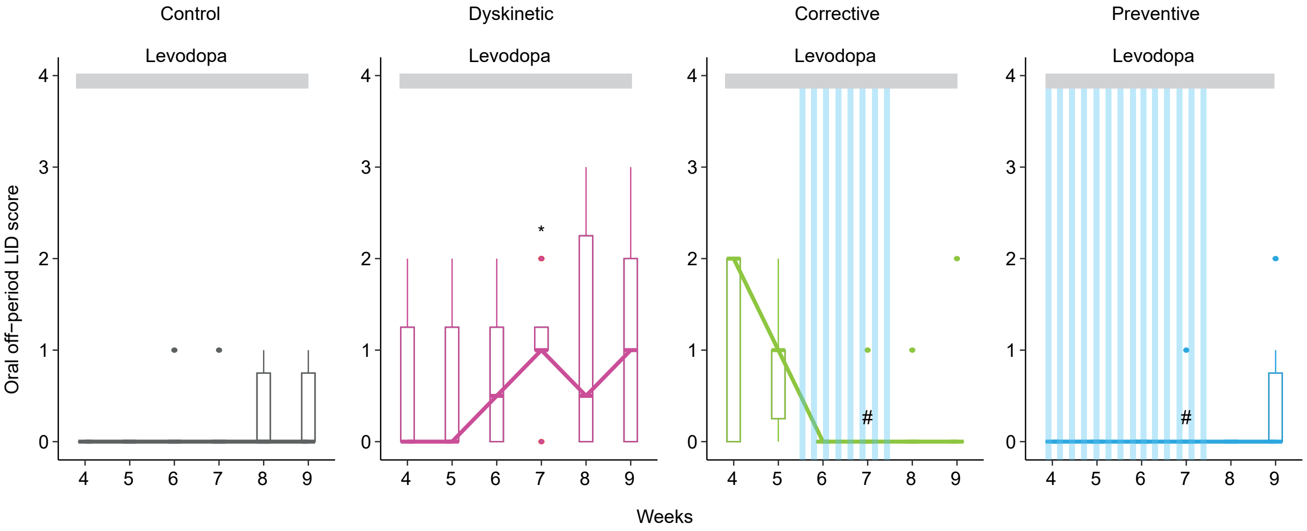

Supplementary Fig. S2 Optogenetic stimulation of CrusII Purkinje cells is sufficient to both reduce and prevent severe orolingual peak-dose dyskinesia. Related to Fig. 1

a Examples of orolingual peak-dose levodopa-induced dyskinesia in L7-ChR2-YFP LID mice chronically treated with levodopa. **b** Boxplot showing the averaged oral LID scores measured over 7 time points starting from the time of levodopa injection (black arrows; 6mg/kg) across the 6 weeks of treatment in SHAM (grey, N=17), LID (magenta, N=19), LID_CORR receiving 2 weeks of Purkinje cell stimulation (green, N=24), and LID_PREV mice receiving 4 weeks of Purkinje cell stimulation (blue, N=18). Boxplots represent the lower and the upper quartiles and horizontal bars in boxplots represent median scores. Vertical lines represent the median +/- std. Isolated points represent outliers of the distribution. Light grey lines: time of levodopa peak-dose effect, 30 minutes after levodopa administration (6mg/kg). Striped blue lines: time of theta-burst stimulation. **c** Boxplot showing the average oral "off-period" LID scores measured 20 minutes before levodopa injection across the 6 weeks of treatment in SHAM (grey, N=8), LID (magenta, N=6), LID_CORR (green, N=6), and LID_PREV mice (blue, N=4). Boxplots represent the lower and the upper quartiles and horizontal bars in boxplots represent median score. Vertical lines represent the median +/- std. Isolated points represent outliers of the distribution. Light grey lines: 6 weeks of levodopa treatment (6mg/kg). Stripped blue lines: weeks of Purkinje cell stimulation. Kruskal-Wallis test with pairwise two-sided Wilcoxon test and Benjamini & Hochberg correction. ***p < 0.001; **p < 0.01; *p < 0.05; * compared to SHAM; # to LID. Source data are provided as a Source Data file. See also Table S1.

Supplementary Figure S3

a

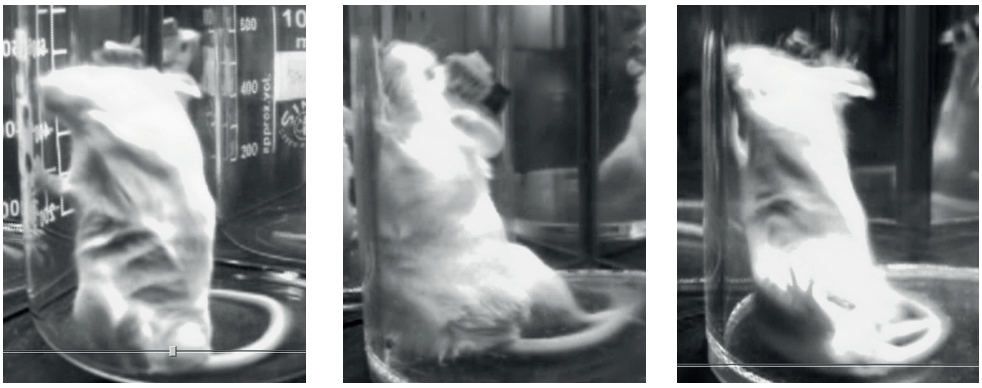

b

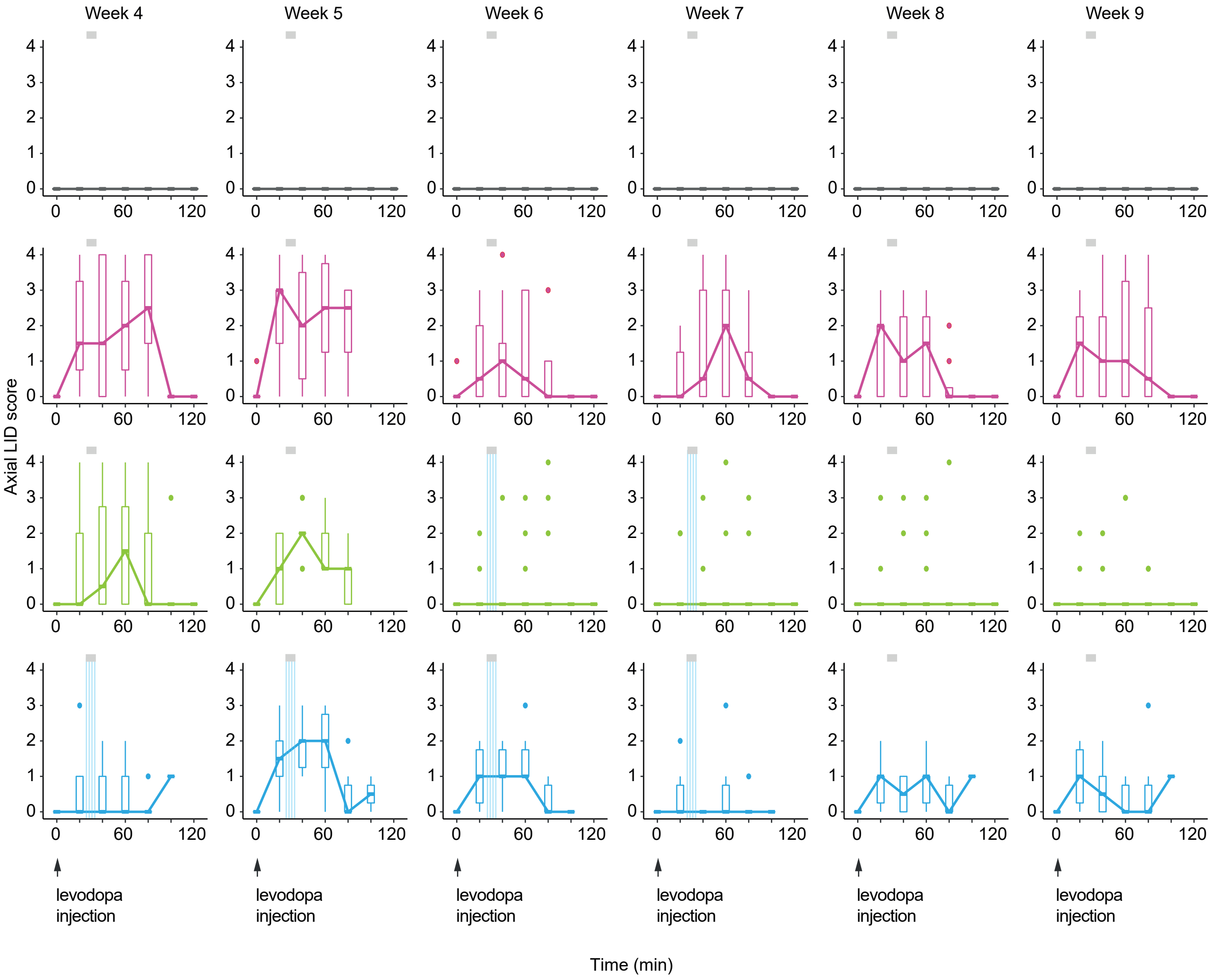

Supplementary Fig. S3 Optogenetic stimulation of CrusII Purkinje cells is not sufficient to completely reduce nor prevent severe axial peak-dose dyskinesia. Related to Fig. 1

a Examples of axial peak-dose levodopa-induced dyskinesia in L7-ChR2-YFP LID mice chronically treated with levodopa. **b** Boxplot showing the averaged axial LID scores measured over 7 time points starting from the time of levodopa injection (black arrows; 6mg/kg) across the 6 weeks of treatment in SHAM (grey, N=12), LID (magenta, N=8), LID_CORR receiving 2 weeks of Purkinje cell stimulation (green, N=14), and LID_PREV mice receiving 4 weeks of Purkinje cell stimulation (blue, N=6). Boxplots represent the lower and the upper quartiles and horizontal bars in boxplots represent median score. Vertical lines represent the median +/- std. Isolated points represent outliers of the distribution. Light grey lines: time of levodopa peak-dose effect, 30 minutes after levodopa administration (6mg/kg). Striped blue lines: time of theta-burst stimulation. Source data are provided as a Source Data file.

Supplementary Figure S4

a

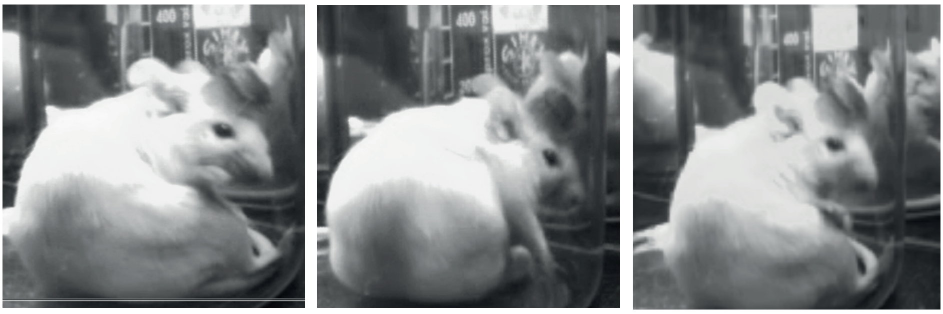

b

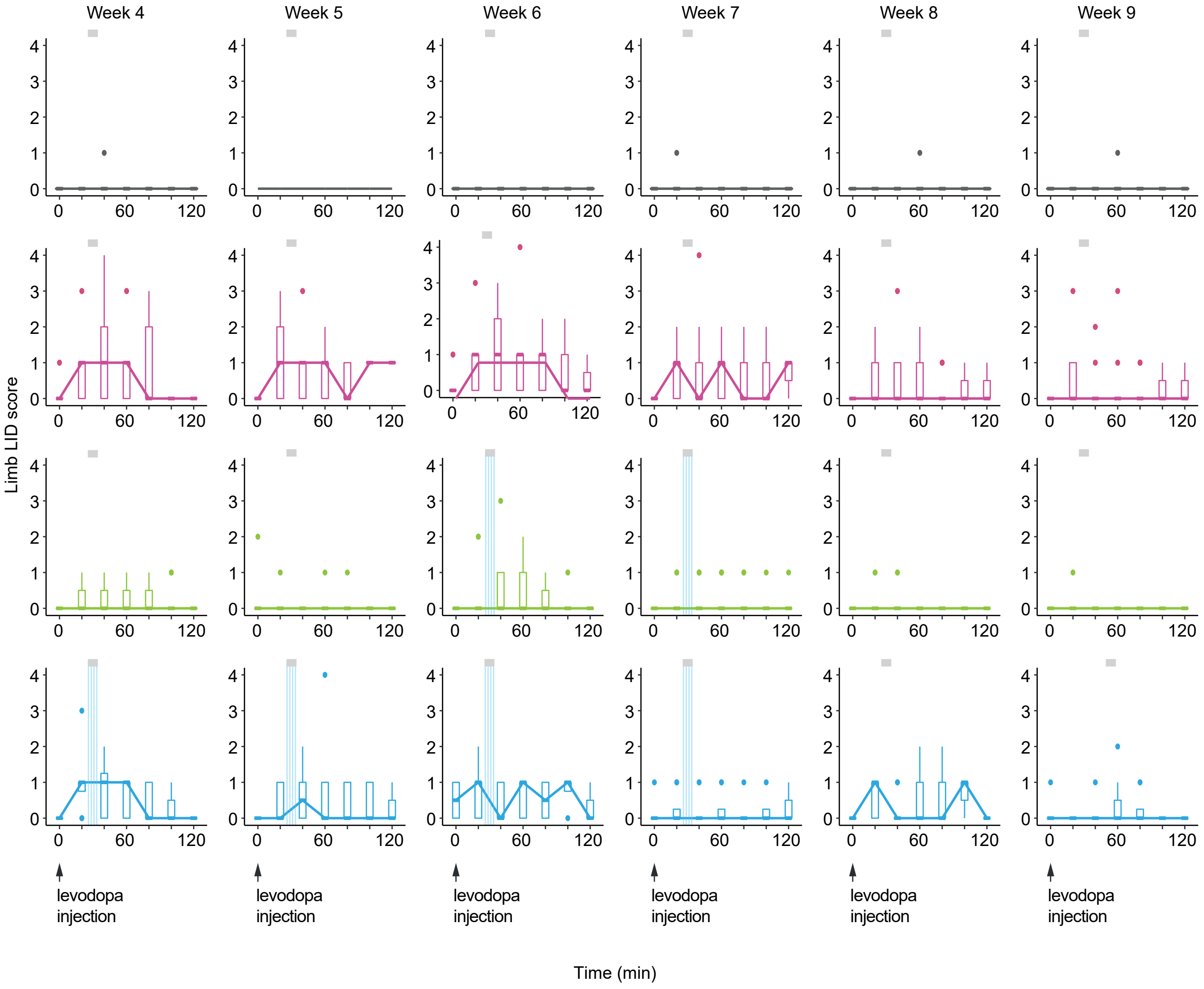

Supplementary Fig. S4 Optogenetic stimulation of CrusII Purkinje cells is not sufficient to completely reduce and prevent severe limb peak-dose dyskinesia. Related to Fig. 1

a Examples of limb peak-dose levodopa-induced dyskinesia in L7-ChR2-YFP LID mice chronically treated with levodopa. **b** Boxplot showing the averaged limb LID scores measured over 7 time points starting from the time of levodopa injection (black arrows; 6mg/kg) across the 6 weeks of treatment in SHAM (grey, N=14), LID (magenta, N=9), LID_CORR receiving 2 weeks of Purkinje cell stimulation (green, N=15), and LID_PREV mice receiving 4 weeks of Purkinje cell stimulation (blue, N=9). Boxplots represent the lower and the upper quartiles and horizontal bars in boxplots represent median score. Vertical lines represent the median +/- std. Isolated points represent outliers of the distribution. Light grey lines: time of levodopa peak-dose effect, 30 minutes after levodopa administration (6mg/kg). Striped blue lines: time of theta-burst stimulation. Source data are provided as a Source Data file.

Supplementary Figure S5

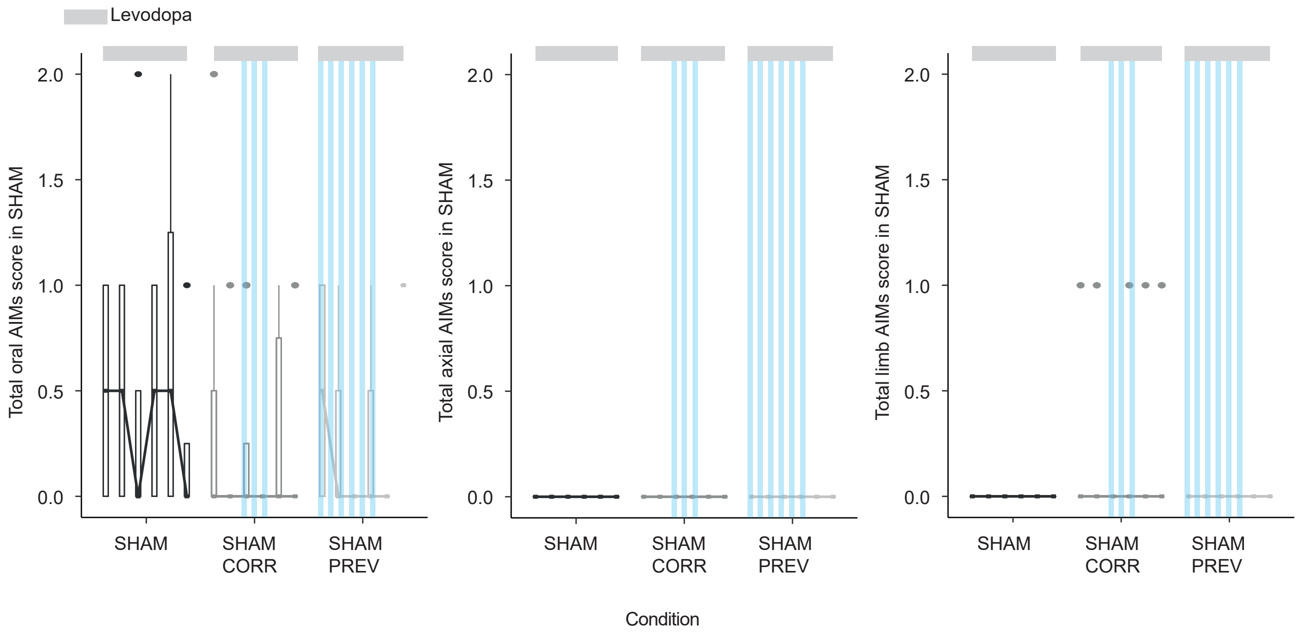

Supplementary Fig. S5 SHAM, either treated with levodopa alone or receiving optogenetic stimulation of CrusII Purkinje cells, do not present oral, axial and limb dyskinesia. Related to Fig. 1

Boxplot showing the sum of oral (left), axial (middle), and limb (right) LID scores measured every 20 minutes (8 time points), starting 20 minutes before levodopa injection to 120 minutes after administration, across the 6 weeks of levodopa treatment (6mg/kg) in SHAM mice (dark grey, SHAM, N=4), SHAM mice treated with levodopa and receiving 2 weeks of Purkinje cell stimulation (grey, SHAM_CORR, N=5), SHAM mice treated with levodopa and receiving 4 weeks of Purkinje cell stimulation (light grey, SHAM_PREV, N=3). Boxplots represents the lower and the upper quartiles. Vertical lines represent the median (horizontal bars) +/- std. Isolated points represent outliers of the distribution. Light grey lines: 6 weeks of levodopa treatment (6mg/kg). Stripped blue lines: time of theta-burst stimulation. Source data are provided as a Source Data file. See also Table S15.

Supplementary Figure S6

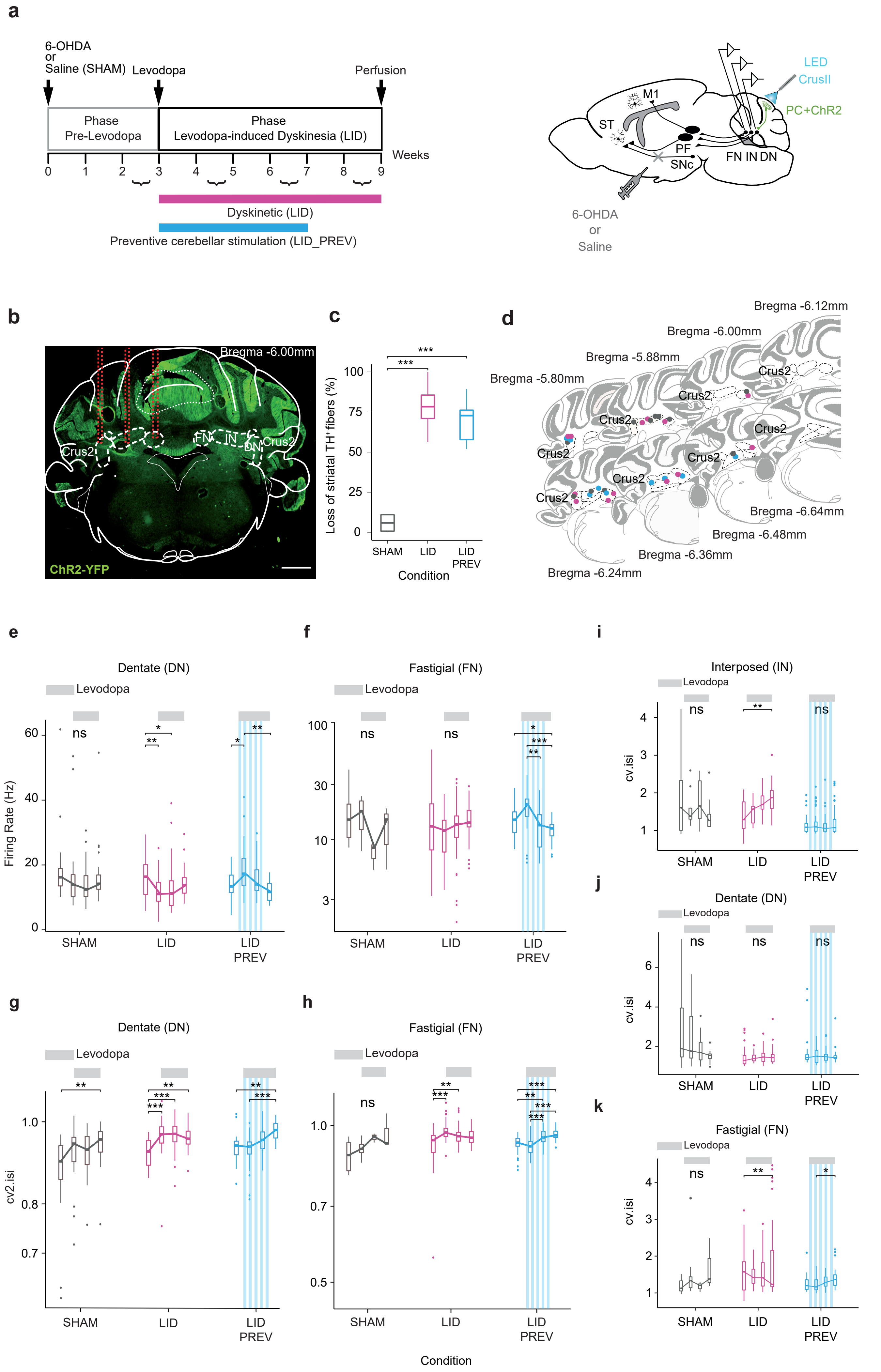

Supplementary Fig. S6. Purkinje cell stimulation does not completely normalize the aberrant activity in the dentate and fastigial cerebellar nuclei. Related to Fig. 2

a *Left*: Experimental timeline. *Right*: Schematic of electrode implantation in the dentate (DN), interposed (IN), and fastigial nuclei (FN), ChR2-YFP in Purkinje cells (PC+ChR2, green) and injection site with 6-OHDA or saline. ST: Striatum; SNc: Substantia nigra *pars compacta*; M1: Primary motor cortex; PF: Parafascicular nucleus of the thalamus; **b** Coronal section from L7-ChR2-YFP mouse showing electrode's trajectory (red lines). Dotted red lines: IN, FN, and DN. Green: ChR2-YFP expression. Scale bars: 0.5 mm (left); 20µm (right). Crus2: Crus2 of the ansiform lobule. **c** Boxplot showing the averaged loss of striatal TH-positive fibers (%) between the lesioned and the intact striatum in SHAM (grey, N=6), LID (magenta, N=4), and LID_PREV (blue, N=5). **d** Schematic of verified recording sites in IN, FN, and DN. **e** Firing rate (Hz) across 9 weeks in DN. Boxplots show the median rate (horizontal bars), over 4 categories of weeks. First boxplot: 2nd and 3rd week of the protocol, second: 4th and 5th weeks, third: 6th and 7th weeks, last: 8th and 9th weeks. **f** Firing rate (Hz) across 9 weeks in FN. **g** Coefficient of variation 2 (cv2.isi) across 9 weeks in DN. **h** Cv2.isi across 9 weeks in FN. **i-k** Coefficient of variation (cv.isi) across 9 weeks in **i** IN, **j** DN, and **k** FN.

Grey = SHAM (DN: N=3, FN: N=3, IN: N=3); Magenta = LID (DN: N=3, FN: N=6, IN: N=3); Blue = LID_PREV (DN: N=3, FN: N=3, IN: N=3). Boxplots represents the lower and the upper quartiles. Vertical lines represent the median +/- std. Isolated points represent outliers of the distribution. Light grey lines: 6 weeks of levodopa (3 boxplots; 6mg/kg). Stripped blue lines: weeks of theta-burst PC stimulation. The order of boxplots in other panels is identical to panel **e**. Welch Anova with two-sided Games Howell post-hoc test

and one-way Anova's with two-sided Tukey post-hoc test based on Levene test. ***p < 0.001; **p < 0.01; *p < 0.05; ns: p > 0.5. Source data are provided as a Source Data file. See also Tables S4, S5, S6, S7.

Supplementary Figure S7

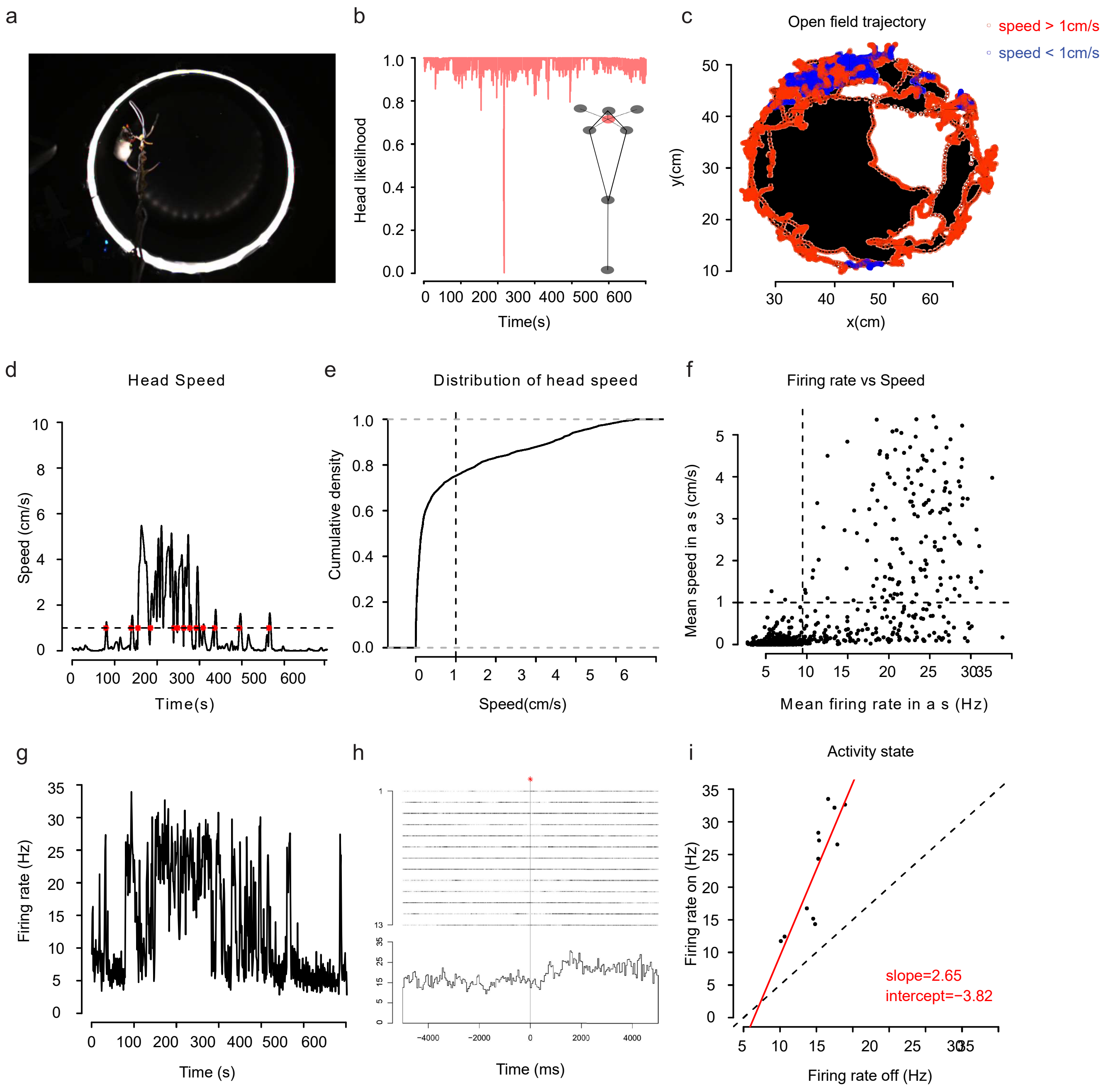

Supplementary Fig. S7 Modulation of the firing rate of the cerebellar nuclei induced by locomotor activity. **Related to Fig. 2**

a Image of the tracking points of interest, represented by different colors (tip of the tail: dark blue; base of the tail: light blue; right ear: dark green; left ear: light green; nose: yellow; right led: dark red; left led: light red) during a recording session in the openfield using Deeplabcut. **b** Evolution of the probability of the head point during the recording. Diagram of the reconstruction of the head point (light red) from the barycenter weighted by the probabilities of the points: nose; right ear; left ear; right led; left led. **c** Path of the head's point as a function of the movement speed. The movement speed is discretized in "off" and "on" periods which are represented by blue and red circles, respectively when the speed is lower and higher than the threshold of 1cm/s. **d** Evolution of the speed of the head's point. The moments when the speed of the head's point crosses the threshold of 1cm/s (dotted line) are reported by a red star. **e** Cumulative distribution of the speed of the head's point. Threshold of 1cm/s: vertical dotted black line. **f** Mean firing rate (Hz) of the cerebellar nuclei (CN) activity as a function of the average velocity defined per second (cm/s). Mean firing rate: vertical dotted line, velocity threshold: horizontal dotted line. **g** Evolution of the average firing rate (Hz) of the CN activity over time. **h** Raster plot of mean CN activity around the transitions from "off" to "on" state of locomotor activity (red stars in D). **i** Relation of the firing rate (Hz) between the locomotor "on" and "off" periods defined per cell (n=12). Dotted line (y=x), straight red line of linear regression of the firing rate of the cells during the "on" periods as a function of the "off" periods. Source data are provided as a Source Data file.

Supplementary Figure S8

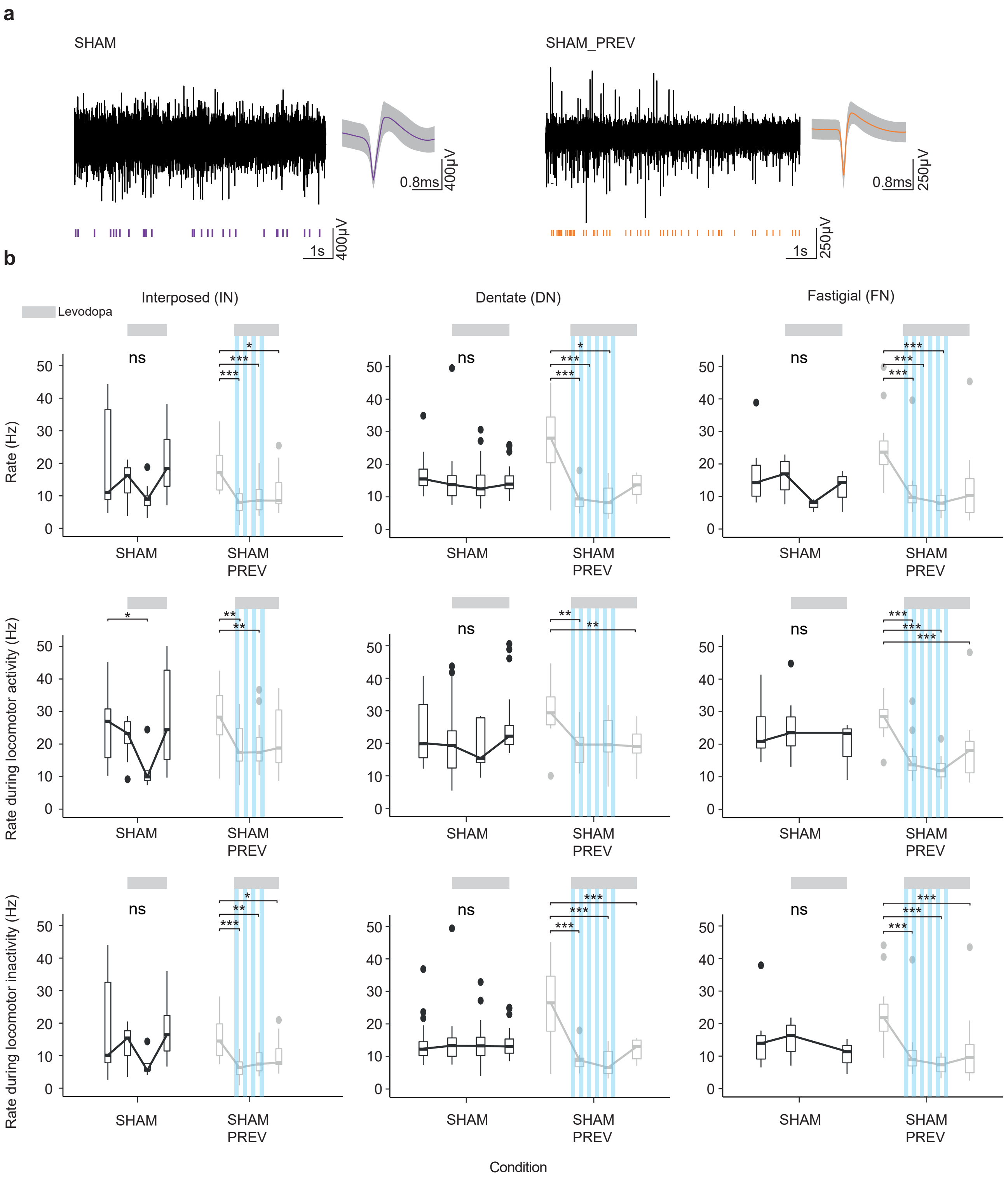

Supplementary Fig. S8 Locomotor activity does not impact the firing rate of the three cerebellar nuclei in SHAM. Related to Fig. 2

a Example of raw traces, and their associated spike shape, recorded in the cerebellar nuclei in SHAM (*left,* purple), treated with levodopa alone and SHAM_PREV (*right,* orange), treated with levodopa and receiving 4 weeks of PC stimulation. **b** *Top*: Firing rate (Hz) over 9 weeks in the three cerebellar nuclei (CN) in SHAM and SHAM_PREV mice: the interposed nucleus (IN, left), the dentate nucleus (DN, middle), and the fastigial nucleus (FN, right). *Middle*: Firing rate (Hz) during periods of locomotor activity ("on") over 9 weeks in CN in SHAM and SHAM_PREV mice: IN (left), DN (middle), and FN (right). *Bottom*: Firing rate (Hz) during periods of locomotor inactivity ("off") over 9 weeks in CN in SHAM and SHAM_PREV mice: IN (left), DN (middle), and FN (right).

Boxplots represent the lower and the upper quartiles and show the median rate (horizontal bars), over 4 categories of weeks. Vertical lines represent the median +/- std. Isolated points represent outliers of the distribution. First boxplot: 2nd and 3rd week of the protocol, second boxplot: 4th and 5th weeks when levodopa treatment started as well as cerebellar stimulation for SHAM_PREV mice, third boxplot: 6th and 7th weeks the last two weeks of cerebellar stimulation, last boxplot: 8th and 9th weeks of the protocol when stimulation stops and long-term effects of cerebellar stimulation are visible. SHAM mice only treated with levodopa represented in dark grey (SHAM, IN: N=1, FN: N=1, DN: N=2); SHAM mice treated with levodopa and receiving 4 weeks of Purkinje cell stimulation represented in grey (SHAM_PREV, IN: N=2, FN: N=2, DN: N=1). Light grey lines: 6 weeks of levodopa (3 boxplots; 6mg/kg). Stripped blue lines: weeks of theta-burst Purkinje cell stimulation. Welch Anova with two-sided Games Howell post-hoc test and one-way Anova's with two-

sided Tukey post-hoc test based on Levene test. ***$p < 0.001$; **$p < 0.01$; *$p < 0.05$; ns: $p > 0.5$. Source data are provided as a Source Data file. See also Table S16.

Supplementary Figure S9

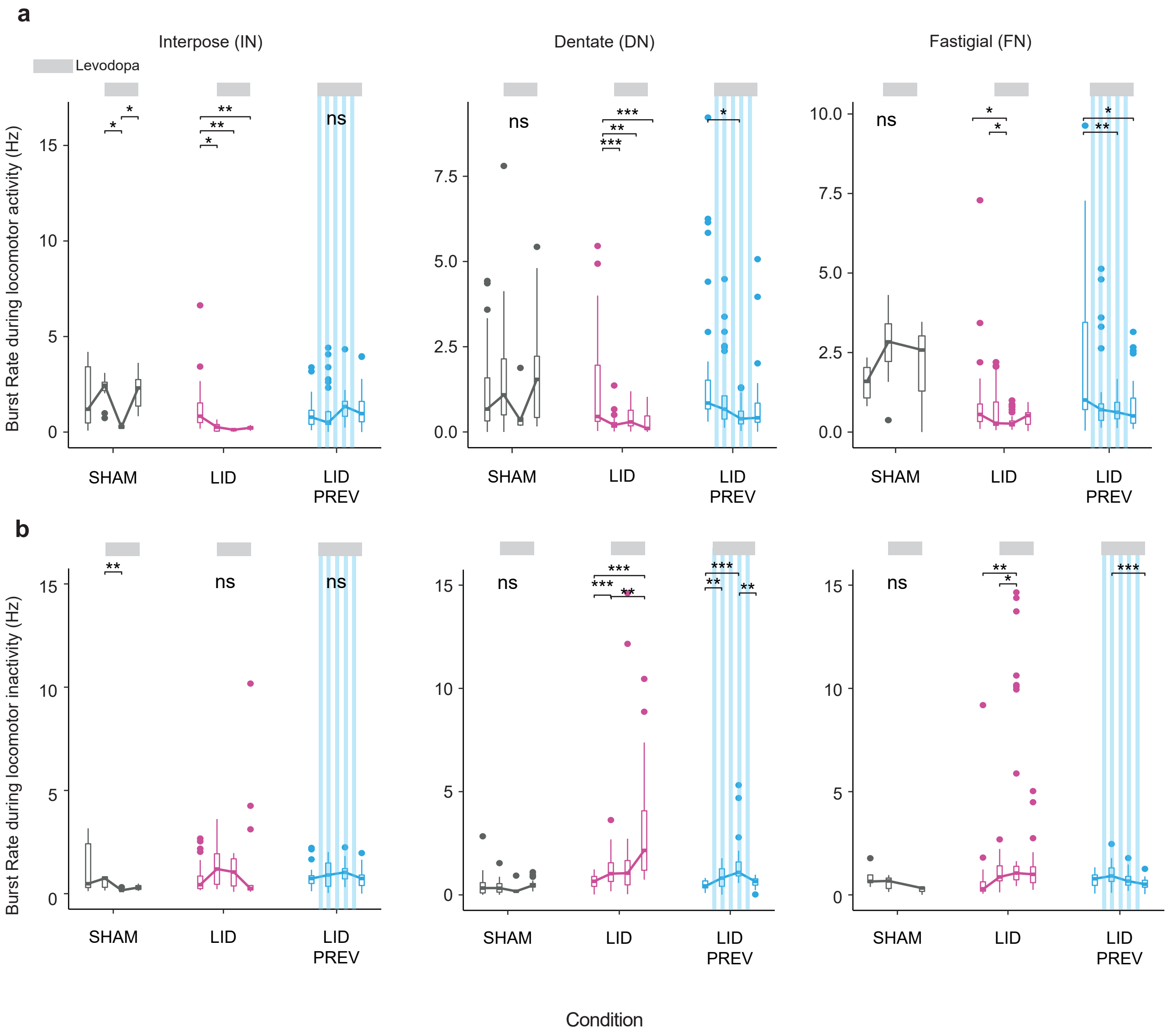

Supplementary Fig. S9 In dyskinetic mice, the burst rate decreases during periods of activity and increases during periods of inactivity. These effects are prevented by cerebellar stimulation in the interposed nucleus. Related to Fig. 2

a Burst rate (Hz) during periods of locomotor activity (“on”) over 9 weeks in the interposed nucleus (IN, left), the dentate nucleus (DN, middle), and the fastigial nucleus (FN, right). Boxplots show the median burst rate (horizontal bars), over 4 categories of weeks. First boxplot: 2nd and 3rd week of the protocol, second boxplot: 4th and 5th weeks when levodopa treatment started, third boxplot: 6th and 7th, last boxplot: 8th and 9th weeks when stimulation stops. Grey = SHAM (IN: N=4, DN: N=3, FN: N=3); Magenta = LID (IN: N=3, DN: N=4, FN: N=6); Blue = LID_PREV (IN: N=3, DN: N=3, FN: N=3). Light grey lines: 6 weeks of levodopa (3 boxplots; 6mg/kg). Stripped blue lines: weeks of theta-burst Purkinje cell stimulation. **b** Burst rate (Hz) during periods of locomotor inactivity (“off”) over 9 weeks in IN (left), DN (middle), and FN (right). Same order of boxplot as panel **a**. Grey = SHAM (IN: N=4, DN: N=3, FN: N=3); Magenta = LID (IN: N=3, DN: N=4, FN: N=6); Blue = LID_PREV (IN: N=3, DN: N=3, FN: N=3). Light grey lines: 6 weeks of levodopa (3 boxplots; 6mg/kg). Stripped blue lines: weeks of theta-burst Purkinje cells’ stimulation.

Boxplots represent the lower and the upper quartiles. Vertical lines represent the median +/- std. Isolated points represent outliers of the distribution. Welch Anova with two-sided Games Howell post-hoc test and one-way Anova’s with two-sided Tukey post-hoc test based on Levene test. ***p < 0.001; **p < 0.01; *p < 0.05; ns: p > 0.5. Source data are provided as a Source Data file. See also Table S17 and S18.

Supplementary Figure S10

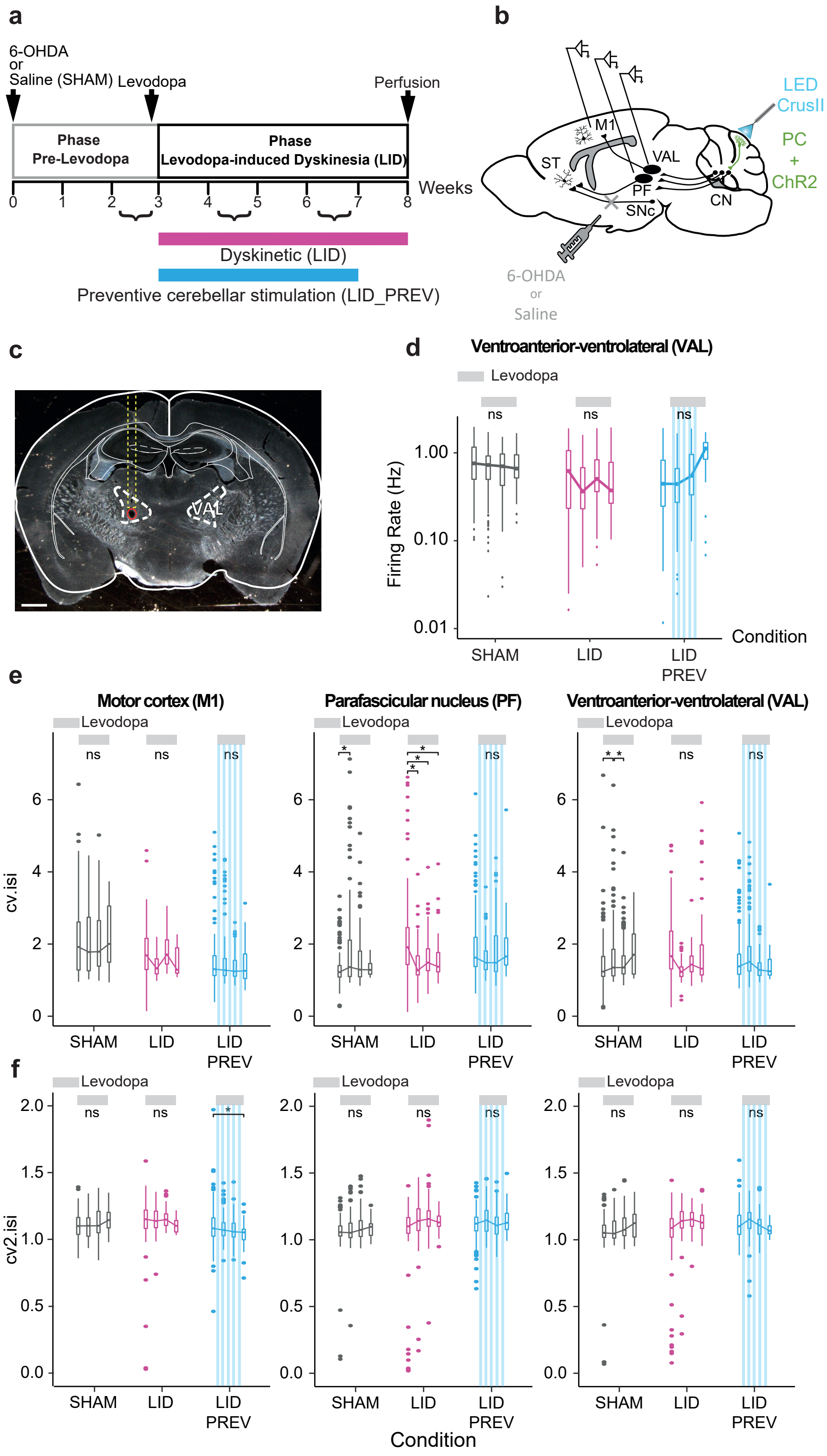

Supplementary Fig. S10 Neither levodopa nor Purkinje cell stimulation affects the firing rate in the cerebellar-region of the ventroanterior-ventrolateral complex of the thalamus. Related to Fig. 3

a Experimental timeline. **b** Schematic of electrode implantation in the cerebellar region of the ventroanterior-ventrolateral complex of the thalamus (VAL), in the primary motor cortex (M1), and in the parafascicular nucleus of the thalamus (PF), ChR2-YFP in Purkinje cells (PC+ChR2, green) and injection site with 6-OHDA or saline. ST: Striatum; SNc: Substantia nigra *pars compacta*; CN: cerebellar nuclei; CrusII: Crus2 of the ansiform lobule. **c** Coronal section from L7-ChR2-YFP mouse showing the electrode's trajectory (yellow dotted line) and the electrolytic lesion (red circle) at the recording site in VAL. Scale bar: 0.5 mm. **d** Firing rate (Hz) across 9 weeks in VAL. Boxplots show the median rate (horizontal bars), over 4 categories of weeks. First boxplot: 2nd and 3rd week of the protocol, second boxplot: 4th and 5th weeks when levodopa treatment started, third boxplot: 6th and 7th weeks, last boxplot represents the 8th week when stimulation stops. Grey = SHAM (N=5); Magenta = LID (N=4); Blue = LID_PREV (N=8). **e** Coefficient of variation (cv.isi) across 9 weeks in M1 (*left*), PF (*middle*), and VAL (*right*). The order of boxplots is identical to panel **d**. **f** Coefficient of variation 2 (cv2.isi) across 9 weeks in M1 (*left*), PF (*middle*), and VAL (*right*). The order of boxplots is identical to panel **d**.

Grey = SHAM (M1: N=5, PF: N=5, and VAL: N=5); Magenta = LID (M1: N=3, PF: N=3, and VAL: N=3); Blue = LID_PREV (M1: N=8, PF: N=8, and VAL: N=8). Boxplots represent the lower and the upper quartiles. Vertical lines represent the median +/- std. Isolated points represent outliers of the distribution. Light grey lines: 6 weeks of levodopa (3 boxplots; 6mg/kg). Stripped blue lines: weeks of theta-burst PC stimulation. One-way

Anova with two-sided Tukey HSD post-hoc test. ***$p < 0.001$; **$p < 0.01$; *$p < 0.05$; ns: $p > 0.5$. Source data are provided as a Source Data file. See also Table S8, S9, S10, and S11.

Table S1: Precision measures, exact p-values, and replicate data relevant to Supplementary Figure 1

Fig	Param	Week	Group1	Group2	Mean1	Mean2	n1	n2	Sum	Adjusted p Value
Fig. Supp1b	Paw touches	1	LID	SHAM	0.64	0.51	8	15	*	0.017
			LID_CORR	SHAM	0.71	0.51	16	15	***	< 0.001
			LID_PREV	SHAM	0.73	0.51	10	15	***	< 0.001
			LID_CORR	LID	0.71	0.64	16	8	ns	0.306
			LID_PREV	LID	0.73	0.64	10	8	ns	0.165
			LID_PREV	LID_CORR	0.73	0.71	10	16	ns	0.939
Fig. Supp1b	Paw touches	2	LID	SHAM	0.70	0.53	8	15	***	< 0.001
			LID_CORR	SHAM	0.70	0.53	17	15	***	< 0.001
			LID_PREV	SHAM	0.78	0.53	10	15	***	< 0.001
			LID_CORR	LID	0.70	0.70	17	8	ns	0.999
			LID_PREV	LID	0.78	0.70	10	8	ns	0.299
			LID_PREV	LID_CORR	0.78	0.70	10	17	ns	0.175
Fig. Supp1b	Paw touches	3	LID	SHAM	0.68	0.53	8	15	*	0.029
			LID_CORR	SHAM	0.70	0.53	17	15	***	< 0.001
			LID_PREV	SHAM	0.76	0.53	10	15	***	< 0.001
			LID_CORR	LID	0.70	0.68	17	8	ns	0.966
			LID_PREV	LID	0.76	0.68	10	8	ns	0.449
			LID_PREV	LID_CORR	0.76	0.70	10	17	ns	0.755

Table S2: Precision measures, exact p-values, and replicate data relevant to Figure 1, and Supplementary Figure 2c

Fig	Param	Week	Group1	Group2	Mean1	Mean2	n1	n2	Sum	Adjusted p Value

Fig. 1c	6OHDA lesion	/	6-OHDA	SHAM	80.93	11.05	40	17	***	< 0.001
Fig. 1e	Oral	4	LID	SHAM	8.84	0.42	19	19	***	< 0.001
			LID_CORR	SHAM	7.96	0.42	23	19	***	< 0.001
			LID_PREV	SHAM	1.59	0.42	17	19	*	0.035
			LID_CORR	LID	7.96	8.84	23	19	ns	0.711
			LID_PREV	LID	1.59	8.84	17	19	###	< 0.001
			LID_PREV	LID_CORR	1.59	7.96	17	23	&&&	< 0.001
Fig. 1e	Oral	5	LID	SHAM	7.79	0.23	19	17	***	< 0.001
			LID_CORR	SHAM	9.20	0.23	15	17	***	< 0.001
			LID_PREV	SHAM	1.83	0.23	18	17	**	0.002
			LID_CORR	LID	9.20	7.79	15	19	ns	0.425
			LID_PREV	LID	1.83	7.79	18	19	###	< 0.001
			LID_PREV	LID_CORR	1.83	9.20	18	15	&&&	< 0.001
Fig. 1e	Oral	6	LID	SHAM	8.31	0.23	19	19	***	< 0.001
			LID_CORR	SHAM	2.71	0.23	24	19	***	< 0.001
			LID_PREV	SHAM	0.94	0.23	18	19	*	0.048
			LID_CORR	LID	2.71	8.31	24	19	###	< 0.001
			LID_PREV	LID	0.94	8.31	18	19	###	< 0.001
			LID_PREV	LID_CORR	0.94	2.71	18	24	&&	0.002
Fig. 1e	Oral	7	LID	SHAM	8.37	0.20	19	15	***	< 0.001
			LID_CORR	SHAM	1.87	0.20	23	15	***	< 0.001

			LID_PREV	SHAM	0.78	0.20	18	15	ns	0.127
			LID_CORR	LID	1.87	8.37	23	19	###	< 0.001
			LID_PREV	LID	0.78	8.37	18	19	###	< 0.001
			LID_PREV	LID_CORR	0.78	1.87	18	23	&	0.010
Fig. 1e	Oral	8	LID	SHAM	8.74	0.35	19	17	***	< 0.001
			LID_CORR	SHAM	0.91	0.35	23	17	ns	0.16
			LID_PREV	SHAM	0.78	0.35	18	17	ns	0.43
			LID_CORR	LID	0.91	8.74	23	19	###	< 0.001
			LID_PREV	LID	0.78	8.74	18	19	###	< 0.001
			LID_PREV	LID_CORR	0.78	0.91	18	23	ns	0.43
Fig. 1e	Oral	9	LID	SHAM	6.42	0.36	19	11	***	< 0.001
			LID_CORR	SHAM	1.31	0.36	16	11	ns	0.053
			LID_PREV	SHAM	1.92	0.36	13	11	**	0.004
			LID_CORR	LID	1.31	6.42	16	19	###	< 0.001
			LID_PREV	LID	1.92	6.42	13	19	###	< 0.001
			LID_PREV	LID_CORR	1.92	1.31	13	16	ns	0.219
Fig. 1f	Axial	4	LID	SHAM	8.12	0	8	12	***	<0.001
			LID_CORR	SHAM	5.07	0	14	12	***	<0.001
			LID_PREV	SHAM	2.40	0	5	12	***	<0.001
			LID_CORR	LID	5.07	8.12	14	8	ns	0.077
			LID_PREV	LID	2.40	8.12	5	8	#	0.018
			LID_PREV	LID_CORR	2.40	5.07	5	14	ns	0.219

Fig. 1f	Axial	5	LID	SHAM	9.00	0	6	9	***	<0.001
			LID_CORR	SHAM	5.40	0	5	9	***	<0.001
			LID_PREV	SHAM	5.83	0	6	9	***	<0.001
			LID_CORR	LID	5.40	9.00	5	6	ns	0.190
			LID_PREV	LID	5.83	9.00	6	6	ns	0.190
			LID_PREV	LID_CORR	5.83	5.40	6	5	ns	0.980
Fig. 1f	Axial	6	LID	SHAM	4.25	0	8	11	***	<0.001
			LID_CORR	SHAM	1.50	0	14	11	**	0.006
			LID_PREV	SHAM	4.00	0	6	11	***	<0.001
			LID_CORR	LID	1.50	4.25	14	8	##	0.002
			LID_PREV	LID	4.00	4.25	6	8	ns	0.610
			LID_PREV	LID_CORR	4.00	1.50	6	14	&&&	<0.001
Fig. 1f	Axial	7	LID	SHAM	4.62	0	8	11	***	<0.001
			LID_CORR	SHAM	1.36	0	14	11	*	0.014
			LID_PREV	SHAM	1.33	0	6	11	**	0.004
			LID_CORR	LID	1.36	4.62	14	8	##	0.002
			LID_PREV	LID	1.33	4.62	6	8	#	0.030
			LID_PREV	LID_CORR	1.33	1.36	6	14	ns	0.586
Fig. 1f	Axial	8	LID	SHAM	4.37	0	8	11	***	<0.001
			LID_CORR	SHAM	1.36	0	14	11	*	0.011
			LID_PREV	SHAM	2.67	0	6	11	***	<0.001
			LID_CORR	LID	1.36	4.37	14	8	##	0.001

			LID_PREV	LID	2.67	4.37	6	8	ns	0.628
			LID_PREV	LID_CORR	2.67	1.36	6	14	&&	0.001
Fig. 1f	Axial	9	LID	SHAM	5.85	0	8	11	***	<0.001
			LID_CORR	SHAM	1.27	0	11	11	**	0.005
			LID_PREV	SHAM	2.83	0	6	11	***	<0.001
			LID_CORR	LID	1.27	5.85	11	8	##	0.001
			LID_PREV	LID	2.83	5.85	6	8	ns	0.246
			LID_PREV	LID_CORR	2.83	1.27	6	11	&	0.021
Fig. 1g	Limb	4	LID	SHAM	3.89	0.07	9	14	***	<0.001
			LID_CORR	SHAM	1.20	0.07	15	14	***	<0.001
			LID_PREV	SHAM	3.12	0.07	8	14	***	<0.001
			LID_CORR	LID	1.20	3.89	15	9	##	0.003
			LID_PREV	LID	3.12	3.89	8	9	ns	0.981
			LID_PREV	LID_CORR	3.12	1.20	8	15	&&	0.002
Fig. 1g	Limb	5	LID	SHAM	3.57	0.09	7	11	***	<0.001
			LID_CORR	SHAM	0.83	0.09	6	11	*	0.034
			LID_PREV	SHAM	2.67	0.09	9	11	***	<0.001
			LID_CORR	LID	0.83	3.57	6	7	##	0.004
			LID_PREV	LID	2.67	3.57	9	7	ns	0.375
			LID_PREV	LID_CORR	2.67	0.83	9	6	&	0.015
Fig. 1g	Limb	6	LID	SHAM	4.22	0	9	13	***	<0.001
			LID_CORR	SHAM	1.53	0	15	13	***	<0.001

			LID_PREV	SHAM	2.89	0	9	13	***	<0.001
			LID_CORR	LID	1.53	4.22	15	9	###	<0.001
			LID_PREV	LID	2.89	4.22	9	9	ns	0.476
			LID_PREV	LID_CORR	2.89	1.53	9	15	&&	0.003
Fig. 1g	Limb	7	LID	SHAM	3.44	0.08	9	12	***	<0.001
			LID_CORR	SHAM	0.86	0.08	14	12	**	0.004
			LID_PREV	SHAM	1.22	0.08	9	12	***	<0.001
			LID_CORR	LID	0.86	3.44	14	9	###	<0.001
			LID_PREV	LID	1.22	3.44	9	9	#	0.018
			LID_PREV	LID_CORR	1.22	0.86	9	14	ns	0.302
Fig. 1g	Limb	8	LID	SHAM	2.22	0.08	9	12	***	<0.001
			LID_CORR	SHAM	0.28	0.08	14	12	ns	0.264
			LID_PREV	SHAM	2.00	0.08	9	12	***	<0.001
			LID_CORR	LID	0.28	2.22	14	9	###	<0.001
			LID_PREV	LID	2.00	2.22	9	9	ns	0.852
			LID_PREV	LID_CORR	2.00	0.28	9	14	&&&	<0.001
Fig. 1g	Limb	9	LID	SHAM	1.89	0.08	9	12	***	<0.001
			LID_CORR	SHAM	0.09	0.08	11	12	ns	0.936
			LID_PREV	SHAM	0.87	0.08	8	12	*	0.014
			LID_CORR	LID	0.09	1.89	11	9	###	<0.001
			LID_PREV	LID	0.87	1.89	8	9	ns	0.291
			LID_PREV	LID_CORR	0.87	0.09	8	11	&	0.018

Supp. Fig2c	Oral	4	LID	SHAM	0.62	0	8	7	ns	0.160
			LID_CORR	SHAM	1.20	0	5	7	ns	0.150
			LID_PREV	SHAM	0	0	5	7	-	-
			LID_CORR	LID	1.20	0.62	5	8	ns	0.370
			LID_PREV	LID	0	0.62	5	8	ns	0.210
			LID_PREV	LID_CORR	0	1.20	5	5	ns	0.160
Supp. Fig2c	Oral	5	LID	SHAM	0.62	0	8	6	ns	0.157
			LID_CORR	SHAM	0.83	0	6	6	ns	0.067
			LID_PREV	SHAM	0	0	6	6	-	-
			LID_CORR	LID	0.83	0.62	6	8	ns	0.575
			LID_PREV	LID	0	0.62	6	8	ns	0.157
			LID_PREV	LID_CORR	0	0.83	6	6	ns	0.067
Supp. Fig2c	Oral	6	LID	SHAM	0.75	0.17	8	6	ns	0.330
			LID_CORR	SHAM	0	0.17	6	6	ns	0.400
			LID_PREV	SHAM	0	0.17	6	6	ns	0.400
			LID_CORR	LID	0	0.75	6	8	ns	0.160
			LID_PREV	LID	0	0.75	6	8	ns	0.160
			LID_PREV	LID_CORR	0	0	6	6	-	-
Supp. Fig2c	Oral	7	LID	SHAM	1.12	0.17	8	6	*	0.028
			LID_CORR	SHAM	0.17	0.17	6	6	ns	1
			LID_PREV	SHAM	0.17	0.17	6	6	ns	1

			LID_CORR	LID	0.17	1.12	6	8	#	0.028
			LID_PREV	LID	0.17	1.12	6	8	#	0.028
			LID_PREV	LID_CORR	0.17	0.17	6	6	ns	1
Supp. Fig2c	Oral	8	LID	SHAM	1.12	0.33	8	6	ns	0.490
			LID_CORR	SHAM	0.17	0.33	6	6	ns	0.590
			LID_PREV	SHAM	0	0.33	6	6	ns	0.350
			LID_CORR	LID	0.17	1.12	6	8	ns	0.350
			LID_PREV	LID	0	1.12	6	8	ns	0.350
			LID_PREV	LID_CORR	0	0.17	6	6	ns	0.49
Supp. Fig2c	Oral	9	LID	SHAM	1.12	0.33	8	6	ns	0.550
			LID_CORR	SHAM	0.33	0.33	6	6	ns	0.900
			LID_PREV	SHAM	0.50	0.33	6	6	ns	0.920
			LID_CORR	LID	0.33	1.12	6	8	ns	0.550
			LID_PREV	LID	0.50	1.12	6	8	ns	0.590
			LID_PREV	LID_CORR	0.50	0.33	6	6	ns	0.900

Table S3: Precision measures, exact p-values, and replicate data relevant to Figure 1c

Fig	Param	Week	Group1	Group2	Mean1	Mean2	n1	n2	Sum	Adjusted p Value
Fig. Supp1c –	Lesion	Phase Pre-Levodopa	LID	SHAM	84.38	11.05	13	17	***	< 0.001
			LID_CORR	SHAM	76.53	11.05	10	17	***	< 0.001
			LID_PREV	SHAM	80.89	11.05	17	17	***	< 0.001
			LID_CORR	LID	76.53	84.38	10	13	ns	0.460

Bottom panel			LID_PREV	LID	80.89	84.38	17	13	ns	0.717
			LID_PREV	LID_CORR	80.89	76.53	17	10	ns	0.952

Table S4: Number of cells and mice in each condition per week for the three deep cerebellar nuclei. Related to Figure 2 and Supplementary Figure 6.

All cell (DCN)	Region	Interposed		Dentate		Fastigial	
Groupe	Weeks	Cells	Mice	Cells	Mice	Cells	Mice
SHAM	W2-W3	n=10	N=1	n=28	N=2	n=7	N=1
	W4-W5	n=11	N=1	n=30	N=2	n=8	N=1
	W6-W7	n=10	N=1	n=24	N=2	n=3	N=1
	W8-W9	n=9	N=1	n=28	N=2	n=3	N=1
LID	W2-W3	n=25	N=3	n=41	N=4	n=41	N=5
	W4-W5	n=8	N=2	n=33	N=4	n=42	N=5
	W6-W7	n=10	N=3	n=24	N=3	n=49	N=6
	W8-W9	n=13	N=3	n=30	N=4	n=27	N=4
LID_PREV	W2-W3	n=47	N=4	n=34	N=3	n=31	N=3
	W4-W5	n=48	N=4	n=26	N=3	n=32	N=3
	W6-W7	n=42	N=4	n=25	N=3	n=32	N=3
	W8-W9	n=43	N=4	n=16	N=3	n=29	N=3

Table S5: Precision measures, exact p-values, and replicate data relevant to Figure 2e and Supplementary Figures 6e and 6f.

**Fig**	**Reg**	**Group**	**Weeks1**	**Weeks2**	**Mean1**	**Mean2**	**n1**	**n2**	**Sum**	**Adjusted p Value**
Fig. 2d	IN	SHAM	W2-W3	W4-W5	19.68 Hz	14.05 Hz	10	11	ns	0.973

			W2-W3	W6-W7	19.68 Hz	9.24 Hz	10	10	ns	0.283
			W2-W3	W8-W9	19.68 Hz	20.48 Hz	10	9	ns	0.841
			W4-W5	W6-W7	14.05 Hz	9.24 Hz	11	10	ns	0.492
			W4-W5	W8-W9	14.05 Hz	20.48 Hz	11	9	ns	0.591
			W6-W7	W8-W9	9.24 Hz	20.48 Hz	10	9	ns	0.063
Fig. 2d	IN	LID	W2-W3	W4-W5	17.35 Hz	9.75 Hz	25	8	ns	0.338
			W2-W3	W6-W7	17.35 Hz	8.50 Hz	25	10	*	0.023
			W2-W3	W8-W9	17.35 Hz	10.64 Hz	25	13	ns	0.147
			W4-W5	W6-W7	9.75 Hz	8.50 Hz	8	10	ns	0.812
			W4-W5	W8-W9	9.75 Hz	10.64 Hz	8	13	ns	0.999
			W6-W7	W8-W9	8.50 Hz	10.64 Hz	10	13	ns	0.818
Fig. 2d	IN	LID	W2-W3	W4-W5	18.74 Hz	18.64 Hz	47	48	ns	0.991
		PREV	W2-W3	W6-W7	18.74 Hz	19.55 Hz	47	42	ns	0.807
			W2-W3	W8-W9	18.74 Hz	18.61 Hz	47	43	ns	0.998
			W4-W5	W6-W7	18.64 Hz	19.55 Hz	48	42	ns	0.636
			W4-W5	W8-W9	18.64 Hz	18.61 Hz	48	43	ns	0.999
			W6-W7	W8-W9	19.55 Hz	18.61Hz	42	43	ns	0.672
Fig.	DN	SHAM	W2-W3	W4-W5	18.31 HZ	16.05 Hz	28	30	ns	0.376
Supp6d			W2-W3	W6-W7	18.31 HZ	14.55 Hz	28	24	ns	0.178
			W2-W3	W8-W9	18.31 HZ	16.56 Hz	28	28	ns	0.791
			W4-W5	W6-W7	16.05 Hz	14.55 Hz	30	24	ns	0.951

			W4-W5	W8-W9	16.05 Hz	16.56 Hz	30	28	ns	0.908
			W6-W7	W8-W9	14.55 Hz	16.56 Hz	24	28	ns	0.656
Fig. Supp6d	DN	LID	W2-W3	W4-W5	16.39 Hz	12.11 Hz	41	33	**	0.007
			W2-W3	W6-W7	16.39 Hz	13.14 Hz	41	24	*	0.033
			W2-W3	W8-W9	16.39 Hz	14.30 Hz	41	30	ns	0.674
			W4-W5	W6-W7	12.11 Hz	13.14 Hz	33	24	ns	0.997
			W4-W5	W8-W9	12.11 Hz	14.30 Hz	33	30	ns	0.210
			W6-W7	W8-W9	13.14 Hz	14.30 Hz	24	30	ns	0.381
Fig. Supp6d	DN	LID PREV	W2-W3	W4-W5	13.77 Hz	18.40 Hz	34	26	*	0.017
			W2-W3	W6-W7	13.77 Hz	15.73 Hz	34	25	ns	0.431
			W2-W3	W8-W9	13.77 Hz	11.77 Hz	34	16	ns	0.586
			W4-W5	W6-W7	18.40 Hz	15.73 Hz	26	25	ns	0.520
			W4-W5	W8-W9	18.40 Hz	11.77 Hz	26	16	**	0.002
			W6-W7	W8-W9	15.73 Hz	11.77 Hz	25	16	ns	0.075
Fig. Supp6f	FN	SHAM	W2-W3	W4-W5	17.24 Hz	15.96 Hz	7	8	ns	1
			W2-W3	W6-W7	17.24 Hz	7.49 Hz	7	3	ns	0.191
			W2-W3	W8-W9	17.24 Hz	12.48 Hz	7	3	ns	0.807
			W4-W5	W6-W7	15.96 Hz	7.49 Hz	8	3	ns	0.193
			W4-W5	W8-W9	15.96 Hz	12.48 Hz	8	3	ns	0.821
			W6-W7	W8-W9	7.49 Hz	12.48 Hz	3	3	ns	0.739
Fig. Supp6f	FN	LID	W2-W3	W4-W5	14.84 Hz	11.20 Hz	41	42	ns	0.467
			W2-W3	W6-W7	14.84 Hz	13.85 Hz	41	49	ns	0.998

			W2-W3	W8-W9	14.84 Hz	14.75 Hz	41	27	ns	0.843
			W4-W5	W6-W7	11.20 Hz	13.85 Hz	42	49	ns	0.527
			W4-W5	W8-W9	11.20 Hz	14.75 Hz	42	27	ns	0.150
			W6-W7	W8-W9	13.85 Hz	14.75 Hz	49	27	ns	0.754
Fig. Supp6d	FN	LID PREV	W2-W3	W4-W5	15.11 Hz	19.29 Hz	31	32	ns	0.095
			W2-W3	W6-W7	15.11 Hz	12.95 Hz	31	32	ns	0.212
			W2-W3	W8-W9	15.11 Hz	11.77 Hz	31	29	*	0.016
			W4-W5	W6-W7	19.29 Hz	12.95 Hz	32	32	**	0.001
			W4-W5	W8-W9	19.29 Hz	11.77 Hz	32	29	***	<0.001
			W6-W7	W8-W9	12.95 Hz	11.77 Hz	32	29	ns	0.960

Table S6: Precision measures, exact p-values, and replicate date relevant to Figure 2f and Supplementary Figures 6g and 6h.

**Fig**	**Reg**	**Group**	**Weeks1**	**Weeks2**	**Mean1**	**Mean2**	**n1**	**n2**	**Sum**	**Adjusted p Value**
Fig. 2e	IN	SHAM	W2-W3	W4-W5	0.882	0.947	10	11	ns	0.166
			W2-W3	W6-W7	0.882	0.959	10	10	ns	0.089
			W2-W3	W8-W9	0.882	0.929	10	9	ns	0.482
			W4-W5	W6-W7	0.947	0.959	11	10	ns	0.982
			W4-W5	W8-W9	0.947	0.929	11	9	ns	0.935
			W6-W7	W8-W9	0.959	0.929	10	9	ns	0.791
Fig. 2e	IN	LID	W2-W3	W4-W5	0.859	1.017	25	8	**	0.003

			W2-W3	W6-W7	0.859	1.023	25	10	***	<0.001
			W2-W3	W8-W9	0.859	1.018	25	13	***	<0.001
			W4-W5	W6-W7	1.017	1.023	8	10	ns	0.999
			W4-W5	W8-W9	1.017	1.018	8	13	ns	1
			W6-W7	W8-W9	1.023	1.018	10	13	ns	0.999
Fig. 2e	IN	LID PREV	W2-W3	W4-W5	0.902	0.909	47	48	ns	0.925
			W2-W3	W6-W7	0.902	0.913	47	42	ns	0.819
			W2-W3	W8-W9	0.902	0.923	47	43	ns	0.316
			W4-W5	W6-W7	0.909	0.913	48	42	ns	0.993
			W4-W5	W8-W9	0.909	0.923	48	43	ns	0.676
			W6-W7	W8-W9	0.913	0.923	42	43	ns	0.845
Fig. Supp6e	DN	SHAM	W2-W3	W4-W5	0.877	0.920	31	32	ns	0.069
			W2-W3	W6-W7	0.877	0.920	31	24	ns	0.093
			W2-W3	W8-W9	0.877	0.943	31	30	**	0.002
			W4-W5	W6-W7	0.920	0.920	32	24	ns	1
			W4-W5	W8-W9	0.920	0.943	32	30	ns	0.552
			W6-W7	W8-W9	0.920	0.943	24	30	ns	0.603
Fig. Supp6e	DN	LID	W2-W3	W4-W5	0.920	0.961	41	33	***	<0.001
			W2-W3	W6-W7	0.920	0.961	41	24	***	<0.001
			W2-W3	W8-W9	0.920	0.956	41	30	**	0.001
			W4-W5	W6-W7	0.961	0.961	33	24	ns	1
			W4-W5	W8-W9	0.961	0.956	33	30	ns	0.947

			W6-W7	W8-W9	0.961	0.956	24	30	ns	0.959
Fig. Supp6e	DN	LID PREV	W2-W3	W4-W5	0.934	0.926	34	26	ns	0.830
			W2-W3	W6-W7	0.934	0.951	34	25	ns	0.284
			W2-W3	W8-W9	0.934	0.975	34	16	**	0.001
			W4-W5	W6-W7	0.926	0.951	26	25	ns	0.070
			W4-W5	W8-W9	0.926	0.975	26	16	***	<0.001
			W6-W7	W8-W9	0.951	0.975	25	16	ns	0.143
Fig. Supp6f	FN	SHAM	W2-W3	W4-W5	0.875	0.903	7	8	ns	0.689
			W2-W3	W6-W7	0.875	0.948	7	3	ns	0.185
			W2-W3	W8-W9	0.875	0.967	7	3	ns	0.069
			W4-W5	W6-W7	0.903	0.948	8	3	ns	0.563
			W4-W5	W8-W9	0.903	0.967	8	3	ns	0.275
			W6-W7	W8-W9	0.948	0.967	3	3	ns	0.966
Fig. Supp6f	FN	LID	W2-W3	W4-W5	0.919	0.977	41	42	***	<0.001
			W2-W3	W6-W7	0.919	0.957	41	49	**	0.004
			W2-W3	W8-W9	0.919	0.950	41	27	ns	0.076
			W4-W5	W6-W7	0.977	0.957	42	49	ns	0.254
			W4-W5	W8-W9	0.977	0.950	42	27	ns	0.154
			W6-W7	W8-W9	0.957	0.950	49	27	ns	0.947
Fig. Supp6f	FN	LID PREV	W2-W3	W4-W5	0.918	0.916	31	32	ns	0.993
			W2-W3	W6-W7	0.918	0.951	31	32	**	0.001
			W2-W3	W8-W9	0.918	0.962	31	29	***	<0.001

			W4-W5	W6-W7	0.916	0.951	32	32	***	<0.001
			W4-W5	W8-W9	0.916	0.962	32	29	***	<0.001
			W6-W7	W8-W9	0.951	0.962	32	29	ns	0.628

Table S7: Precision measures, exact p-values, and replicate data relevant to Supplementary Figure 6i, j, and k

Fig	Reg	Group	Weeks1	Weeks2	Mean1	Mean2	n1	n2	Sum	Adjusted p Value
Fig. Supp6i	IN	SHAM	W2-W3	W4-W5	1.148	1.600	10	11	ns	0.770
			W2-W3	W6-W7	1.148	1.778	10	10	ns	0.996
			W2-W3	W8-W9	1.148	1.433	10	9	ns	0.556
			W4-W5	W6-W7	1.600	1.778	11	10	ns	0.884
			W4-W5	W8-W9	1.600	1.433	11	9	ns	0.976
			W6-W7	W8-W9	1.778	1.433	10	9	ns	0.693
Fig. Supp6i	IN	LID	W2-W3	W4-W5	1.380	1.490	25	8	ns	0.921
			W2-W3	W6-W7	1.380	1.725	25	10	ns	0.149
			W2-W3	W8-W9	1.380	1.890	25	13	**	0.005
			W4-W5	W6-W7	1.490	1.725	8	10	ns	0.654
			W4-W5	W8-W9	1.490	1.890	8	13	ns	0.173
			W6-W7	W8-W9	1.725	1.890	10	13	ns	0.796
Fig. Supp6i	IN	LID PREV	W2-W3	W4-W5	1.138	1.198	47	48	ns	0.757
			W2-W3	W6-W7	1.138	1.139	47	42	ns	1

			W2-W3	W8-W9	1.138	1.247	47	43	ns	0.296
			W4-W5	W6-W7	1.198	1.139	48	42	ns	0.784
			W4-W5	W8-W9	1.198	1.247	48	43	ns	0.852
			W6-W7	W8-W9	1.139	1.247	42	43	ns	0.331
Fig. Supp6j	DN	SHAM	W2-W3	W4-W5	2.915	2.469	31	32	ns	0.619
			W2-W3	W6-W7	2.915	1.852	31	24	ns	1
			W2-W3	W8-W9	2.915	1.511	31	30	ns	0.645
			W4-W5	W6-W7	2.469	1.852	32	24	ns	0.754
			W4-W5	W8-W9	2.469	1.511	32	30	ns	0.993
			W6-W7	W8-W9	1.852	1.511	24	30	ns	0.725
Fig. Supp6j	DN	LID	W2-W3	W4-W5	1.435	1.441	41	33	ns	1
			W2-W3	W6-W7	1.435	1.540	41	24	ns	0.754
			W2-W3	W8-W9	1.435	1.522	41	30	ns	0.819
			W4-W5	W6-W7	1.441	1.540	33	24	ns	0.809
			W4-W5	W8-W9	1.441	1.522	33	30	ns	0.868
			W6-W7	W8-W9	1.540	1.522	24	30	ns	0 .998
Fig. Supp6j	DN	LID PREV	W2-W3	W4-W5	1.648	1.555	34	26	ns	0.926
			W2-W3	W6-W7	1.648	1.542	34	25	ns	0.898
			W2-W3	W8-W9	1.648	1.580	34	16	ns	0.980
			W4-W5	W6-W7	1.555	1.542	26	25	ns	1
			W4-W5	W8-W9	1.555	1.580	26	16	ns	0.999
			W6-W7	W8-W9	1.542	1.580	25	16	ns	0.997

Fig. Supp6k	FN	SHAM	W2-W3	W4-W5	1.196	1.577	7	8	ns	0.619
			W2-W3	W6-W7	1.196	1.173	7	3	ns	1
			W2-W3	W8-W9	1.196	1.686	7	3	ns	0.645
			W4-W5	W6-W7	1.577	1.173	8	3	ns	0.754
			W4-W5	W8-W9	1.577	1.686	8	3	ns	0.993
			W6-W7	W8-W9	1.173	1.686	3	3	ns	0.725
Fig. Supp6k	FN	LID	W2-W3	W4-W5	1.585	1.471	41	42	ns	0.921
			W2-W3	W6-W7	1.585	1.583	41	49	ns	0.149
			W2-W3	W8-W9	1.585	1.902	41	27	**	0.005
			W4-W5	W6-W7	1.471	1.583	42	49	ns	0.654
			W4-W5	W8-W9	1.471	1.902	42	27	ns	0.172
			W6-W7	W8-W9	1.583	1.902	49	27	ns	0.796
Fig. Supp6k	FN	LID PREV	W2-W3	W4-W5	1.257	1.237	31	32	ns	0.984
			W2-W3	W6-W7	1.257	1.312	31	32	ns	0.773
			W2-W3	W8-W9	1.257	1.406	31	29	ns	0.058
			W4-W5	W6-W7	1.237	1.312	32	32	ns	0.546
			W4-W5	W8-W9	1.237	1.406	32	29	*	0.022
			W6-W7	W8-W9	1.312	1.406	32	29	ns	0.370

Table S8: Number of cells and mice in each condition per week in the motor cortex (M1), the parafascicular nucleus (PF) of the thalamus and the ventroanterior-ventrolateral complex (VAL) of the thalamus. Related to Figures 3c, 3d and Supplementary Figure 10d.

All cell	Region	M1		PF		VAL	
Groupe	Weeks	Cells	Mice	Cells	Mice	Cells	Mice
SHAM	W2-W3	n=169	N=5	n=125	N=5	n=150	N=5
	W4-W5	n=137	N=5	n=107	N=5	n=136	N=5
	W6-W7	n=123	N=5	n=104	N=5	n=161	N=5
	W8	n=37	N=3	n=16	N=2	n=46	N=3
LID	W2-W3	n=107	N=4	n=83	N=4	n=135	N=4
	W4-W5	n=73	N=3	n=80	N=3	n=81	N=3
	W6-W7	n=65	N=3	n=83	N=3	n=105	N=3
	W8	n=34	N=3	n=32	N=3	n=46	N=3
LID_PREV	W2-W3	n=221	N=8	n=159	N=8	n=164	N=8
	W4-W5	n=172	N=8	n=168	N=8	n=154	N=8
	W6-W7	n=120	N=8	n=131	N=8	n=123	N=8
	W8	n=41	N=4	n=37	N=5	n=29	N=5

Table S9: Precision measures, exact p-values, and replicate date relevant to Figures 3c, 3d and Supplementary Figure 10d.

**Fig**	**Reg**	**Group**	**Weeks1**	**Weeks2**	**Mean1**	**Mean2**	**n1**	**n2**	**Sum**	**Adjusted p Value**
Fig.3c	M1	SHAM	W2-W3	W4-W5	1.01 Hz	0.93 Hz	169	137	ns	0.989
			W2-W3	W6-W7	1.01 Hz	0.80 Hz	169	123	ns	0.790
			W2-W3	W8	1.01 Hz	0.85 Hz	169	37	ns	0.880
			W4-W5	W6-W7	0.93 Hz	0.80 Hz	123	137	ns	0.934
			W4-W5	W8	0.93 Hz	0.85 Hz	123	37	ns	0.772
			W6-W7	W8	0.80 Hz	0.85 Hz	137	37	ns	0.491
Fig.3c	M1	LID	W2-W3	W4-W5	0.30 Hz	0.83 Hz	107	73	***	<0.001

			W2-W3	W6-W7	0.30 Hz	1.16 Hz	107	65	***	<0.001
			W2-W3	W8	0.30 Hz	1.39 Hz	107	34	***	<0.001
			W4-W5	W6-W7	0.83 Hz	1.16 Hz	73	65	ns	0.567
			W4-W5	W8	0.83 Hz	1.39 Hz	73	34	ns	0.120
			W6-W7	W8	1.16 Hz	1.39 Hz	65	34	ns	0.768
Fig.3c	M1	LID	W2-W3	W4-W5	0.56 Hz	0.70 Hz	221	172	ns	0.594
		PREV	W2-W3	W6-W7	0.56 Hz	0.85 Hz	221	120	ns	0.156
			W2-W3	W8	0.56 Hz	0.56 Hz	221	41	ns	0.574
			W4-W5	W6-W7	0.70 Hz	0.85 Hz	172	120	ns	0.833
			W4-W5	W8	0.70 Hz	0.56 Hz	172	41	ns	0.983
			W6-W7	W8	0.85 Hz	0.56 Hz	120	41	ns	0.994
Fig.3d	PF	SHAM	W2-W3	W4-W5	0.91 Hz	0.71 Hz	125	107	ns	0.662
			W2-W3	W6-W7	0.91 Hz	0.68 Hz	125	104	ns	0.906
			W2-W3	W8	0.91 Hz	0.60 Hz	125	16	ns	0.766
			W4-W5	W6-W7	0.71 Hz	0.68 Hz	107	104	ns	0.966
			W4-W5	W8	0.71 Hz	0.60 Hz	107	16	ns	0.994
			W6-W7	W8	0.68 Hz	0.60 Hz	104	16	ns	0.946
Fig.3d	PF	LID	W2-W3	W4-W5	1.06 Hz	0.34 Hz	83	80	ns	0.053
			W2-W3	W6-W7	1.06 Hz	0.31 Hz	83	83	*	0.025
			W2-W3	W8	1.06 Hz	0.40 Hz	83	32	ns	0.102
			W4-W5	W6-W7	0.34 Hz	0.31 Hz	80	83	ns	0.993
			W4-W5	W8	0.34 Hz	0.40 Hz	80	32	ns	1

			W6-W7	W8	0.31 Hz	0.40 Hz	83	32	ns	0.992
Fig.3d	PF	LID PREV	W2-W3	W4-W5	1.11 Hz	0.58 Hz	159	168	ns	0.086
			W2-W3	W6-W7	1.11 Hz	0.72 Hz	159	131	ns	0.146
			W2-W3	W8	1.11 Hz	0.74 Hz	159	37	ns	0.672
			W4-W5	W6-W7	0.58 Hz	0.72 Hz	168	131	ns	0.996
			W4-W5	W8	0.58 Hz	0.74 Hz	168	37	ns	0.906
			W6-W7	W8	0.72 Hz	0.74 Hz	131	37	ns	0.958
Fig. Supp10d	VAL	SHAM	W2-W3	W4-W5	0.92 Hz	0.72 Hz	150	136	ns	0.298
			W2-W3	W6-W7	0.92 Hz	0.72 Hz	150	161	ns	0.411
			W2-W3	W8	0.92 Hz	0.75 Hz	150	46	ns	0.890
			W4-W5	W6-W7	0.72 Hz	0.72 Hz	136	161	ns	0.996
			W4-W5	W8	0.72 Hz	0.75 Hz	136	46	ns	0.908
			W6-W7	W8	0.72 Hz	0.75 Hz	161	46	ns	0.957
Fig. Supp10d	VAL	LID	W2-W3	W4-W5	0.92 Hz	0.48 Hz	135	81	ns	0.856
			W2-W3	W6-W7	0.92 Hz	0.61 Hz	135	105	ns	0.990
			W2-W3	W8	0.92 Hz	0.66 Hz	135	46	ns	0.964
			W4-W5	W6-W7	0.48 Hz	0.61 Hz	81	105	ns	0.967
			W4-W5	W8	0.48 Hz	0.66 Hz	81	46	ns	0.992
			W6-W7	W8	0.61 Hz	0.66 Hz	105	46	ns	0.998
Fig. Supp10d	VAL	LID PREV	W2-W3	W4-W5	0.66 Hz	0.52 Hz	164	154	ns	0.952
			W2-W3	W6-W7	0.66 Hz	0.70 Hz	164	123	ns	0.929
			W2-W3	W8	0.66 Hz	1.06 Hz	164	29	ns	0.910

			W4-W5	W6-W7	0.52 Hz	0.70 Hz	154	123	ns	0.665
			W4-W5	W8	0.52 Hz	1.06 Hz	154	29	ns	0.685
			W6-W7	W8	0.70 Hz	1.06 Hz	123	29	ns	0.999

Table S10: Precision measures, exact p-values, and replicate date relevant to Supplementary Figure 10e.

**Fig**	**Reg**	**Group**	**Weeks1**	**Weeks2**	**Mean1**	**Mean2**	**n1**	**n2**	**Sum**	**Adjusted p Value**
Fig. Supp10e – left panel	M1	SHAM	W2-W3	W4-W5	2.093	2.075	169	137	ns	0.936
			W2-W3	W6-W7	2.093	2.053	169	123	ns	0.999
			W2-W3	W8-W9	2.093	2.229	169	37	ns	1
			W4-W5	W6-W7	2.075	2.053	123	137	ns	0.891
			W4-W5	W8-W9	2.075	2.229	123	37	ns	0.978
			W6-W7	W8-W9	2.053	2.229	137	37	ns	0.999
Fig. Supp10e – left panel	M1	LID	W2-W3	W4-W5	1.795	1.394	107	73	ns	0.313
			W2-W3	W6-W7	1.795	1.847	107	65	ns	1
			W2-W3	W8-W9	1.795	1.504	107	34	ns	0.741
			W4-W5	W6-W7	1.394	1.847	73	65	ns	0.339
			W4-W5	W8-W9	1.394	1.504	73	34	ns	0.919
			W6-W7	W8-W9	1.847	1.504	65	34	ns	0.752
Fig. Supp10e	M1	LID PREV	W2-W3	W4-W5	1.523	1.527	221	172	ns	0.997
			W2-W3	W6-W7	1.523	1.367	221	120	ns	0.863

– left panel			W2-W3	W8-W9	1.523	1.429	221	41	ns	0.982
			W4-W5	W6-W7	1.527	1.367	172	120	ns	0.763
			W4-W5	W8-W9	1.527	1.429	172	41	ns	0.996
			W6-W7	W8-W9	1.367	1.429	120	41	ns	0.776
Fig. Supp10e – middle panel	PF	SHAM	W2-W3	W4-W5	1.381	1.919	125	107	*	0.041
			W2-W3	W6-W7	1.381	1.553	125	104	ns	0.933
			W2-W3	W8-W9	1.381	1.349	125	16	ns	1
			W4-W5	W6-W7	1.919	1.553	107	104	ns	0.169
			W4-W5	W8-W9	1.919	1.349	107	16	ns	0.393
			W6-W7	W8-W9	1.553	1.349	104	16	ns	0.987
Fig. Supp10e – middle panel	PF	LID	W2-W3	W4-W5	2.208	1.458	83	80	*	0.011
			W2-W3	W6-W7	2.208	1.653	83	83	*	0.033
			W2-W3	W8-W9	2.208	1.625	83	32	*	0.048
			W4-W5	W6-W7	1.458	1.653	80	83	ns	0.988
			W4-W5	W8-W9	1.458	1.625	80	32	ns	0.998
			W6-W7	W8-W9	1.653	1.625	83	32	ns	0.999
Fig. Supp10e – middle panel	PF	LID PREV	W2-W3	W4-W5	1.944	1.583	159	168	ns	0.781
			W2-W3	W6-W7	1.944	1.772	159	131	ns	0.938
			W2-W3	W8-W9	1.944	1.854	159	37	ns	0.949
			W4-W5	W6-W7	1.583	1.772	168	131	ns	0.984
			W4-W5	W8-W9	1.583	1.854	168	37	ns	0.554
			W6-W7	W8-W9	1.772	1.854	131	37	ns	0.741

Fig. Supp10e – right panel	VAL	SHAM	W2-W3	W4-W5	1.501	1.683	150	136	*	0.018
			W2-W3	W6-W7	1.501	1.523	150	161	ns	1
			W2-W3	W8-W9	1.501	1.785	150	46	ns	0.723
			W4-W5	W6-W7	1.683	1.523	136	161	*	0.013
			W4-W5	W8-W9	1.683	1.785	136	46	ns	0.621
			W6-W7	W8-W9	1.523	1.785	161	46	ns	0.667
Fig. Supp10e – right panel	VAL	LID	W2-W3	W4-W5	1.851	1.257	135	81	ns	0.397
			W2-W3	W6-W7	1.851	1.486	135	105	ns	0.785
			W2-W3	W8-W9	1.851	1.947	135	46	ns	0.999
			W4-W5	W6-W7	1.257	1.486	81	105	ns	0.930
			W4-W5	W8-W9	1.257	1.947	81	46	ns	0.377
			W6-W7	W8-W9	1.486	1.947	105	46	ns	0.743
Fig. Supp10e – right panel	VAL	LID PREV	W2-W3	W4-W5	1.652	1.793	164	154	ns	1
			W2-W3	W6-W7	1.652	1.370	164	123	ns	0.838
			W2-W3	W8-W9	1.652	1.418	164	29	ns	0.893
			W4-W5	W6-W7	1.793	1.370	154	123	ns	0.820
			W4-W5	W8-W9	1.793	1.418	154	29	ns	0.881
			W6-W7	W8-W9	1.370	1.418	123	29	ns	1

Table S11: Precision measures, exact p-values, and replicate date relevant to Supplementary Figure 10f.

Fig	Reg	Group	Weeks1	Weeks2	Mean1	Mean2	n1	n2	Sum	Adjusted p Value
Fig. Supp10f – left panel	M1	SHAM	W2-W3	W4-W5	1.105	1.101	169	137	ns	1
			W2-W3	W6-W7	1.105	1.129	169	123	ns	0.924
			W2-W3	W8-W9	1.105	1.150	169	37	ns	1
			W4-W5	W6-W7	1.101	1.129	123	137	ns	0.931
			W4-W5	W8-W9	1.101	1.150	123	37	ns	1
			W6-W7	W8-W9	1.129	1.150	137	37	ns	0.962
Fig. Supp10f – left panel	M1	LID	W2-W3	W4-W5	1.131	1.149	107	73	ns	0.999
			W2-W3	W6-W7	1.131	1.154	107	65	ns	1
			W2-W3	W8-W9	1.131	1.102	107	34	ns	0.380
			W4-W5	W6-W7	1.149	1.154	73	65	ns	0.997
			W4-W5	W8-W9	1.149	1.102	73	34	ns	0.481
			W6-W7	W8-W9	1.154	1.102	65	34	ns	0.395
Fig. Supp10f – left panel	M1	LID PREV	W2-W3	W4-W5	1.096	1.085	221	172	ns	0.820
			W2-W3	W6-W7	1.096	1.071	221	120	ns	0.204
			W2-W3	W8-W9	1.096	1.038	221	41	*	0.033
			W4-W5	W6-W7	1.085	1.071	172	120	ns	0.689
			W4-W5	W8-W9	1.085	1.038	172	41	ns	0.133
			W6-W7	W8-W9	1.071	1.038	120	41	ns	0.497
Fig. Supp10f	PF	SHAM	W2-W3	W4-W5	1.056	1.082	125	107	ns	0.848
			W2-W3	W6-W7	1.056	1.101	125	104	ns	0.590

– middle panel			W2-W3	W8-W9	1.056	1.092	125	16	ns	0.991
			W4-W5	W6-W7	1.082	1.101	107	104	ns	0.970
			W4-W5	W8-W9	1.082	1.092	107	16	ns	0.997
			W6-W7	W8-W9	1.101	1.092	104	16	ns	0.966
Fig. Supp10f – middle panel	PF	LID	W2-W3	W4-W5	1.035	1.131	83	80	ns	0.476
			W2-W3	W6-W7	1.035	1.168	83	83	ns	0.138
			W2-W3	W8-W9	1.035	1.135	83	32	ns	0.862
			W4-W5	W6-W7	1.131	1.168	80	83	ns	0.877
			W4-W5	W8-W9	1.131	1.135	80	32	ns	0.986
			W6-W7	W8-W9	1.168	1.135	83	32	ns	0.791
Fig. Supp10f – middle panel	PF	LID PREV	W2-W3	W4-W5	1.115	1.149	159	168	ns	0.063
			W2-W3	W6-W7	1.115	1.114	159	131	ns	0.989
			W2-W3	W8-W9	1.115	1.149	159	37	ns	0.798
			W4-W5	W6-W7	1.149	1.114	168	131	ns	0.148
			W4-W5	W8-W9	1.149	1.149	168	37	ns	0.872
			W6-W7	W8-W9	1.114	1.149	131	37	ns	0.899
Fig. Supp10f – right panel	VAL	SHAM	W2-W3	W4-W5	1.052	1.083	150	136	ns	0.315
			W2-W3	W6-W7	1.052	1.100	150	161	ns	0.320
			W2-W3	W8-W9	1.052	1.120	150	46	ns	0.377
			W4-W5	W6-W7	1.083	1.100	136	161	ns	1
			W4-W5	W8-W9	1.083	1.120	136	46	ns	0.994
			W6-W7	W8-W9	1.100	1.120	161	46	ns	0.993

Fig. Supp10f – right panel	VAL	LID	W2-W3	W4-W5	1.048	1.129	135	81	ns	0.472
			W2-W3	W6-W7	1.048	1.150	135	105	ns	0.268
			W2-W3	W8-W9	1.048	1.140	135	46	ns	0.218
			W4-W5	W6-W7	1.129	1.150	81	105	ns	0.987
			W4-W5	W8-W9	1.129	1.140	81	46	ns	0.957
			W6-W7	W8-W9	1.150	1.140	105	46	ns	0.998
Fig. Supp10f – right panel	VAL	LID PREV	W2-W3	W4-W5	1.118	1.140	164	154	ns	0.258
			W2-W3	W6-W7	1.118	1.112	164	123	ns	0.984
			W2-W3	W8-W9	1.118	1.076	164	29	ns	0.920
			W4-W5	W6-W7	1.140	1.112	154	123	ns	0.481
			W4-W5	W8-W9	1.140	1.076	154	29	ns	0.222
			W6-W7	W8-W9	1.112	1.076	123	29	ns	0.811

Table S12: Precision measures, exact p-values, and replicate date relevant to Figure 5.

**Fig**	**Param**	**Group1**	**Group2**	**Mean1**	**Mean2**	**n1**	**n2**	**Sum**	**Adjusted p Value**
Fig.5e	% of cells expressing DREADDs	CTL	LID_PREV Saline	10.82%	24.18%	3	5	***	<0.001
		CTL	LID_PREV CNO	10.82%	22.72%	3	6	***	<0.001
		LID_PREV	LID_PREV	24.183%	22.72%	5	6	ns	0.714

		Saline	CNO						
Fig.5f	Oral LID	LID_PREV<br><br>Saline	LID_PREV<br><br>CNO	2.4	9.17	5	7	***	< 0.001

Table S13: STDP in D2$^-$ and D2$^+$ MSN from SHAM, SHAM_PREV, LID and LID_PREV mice. Related to Figure 6.

Group	D2$^-$ MSN	D2$^+$ MSN
SHAM (N=5)	149 ± 2, p<0.0001 (n=11)	142 ± 4, p<0.0001 (n=10)
SHAM_PREV (N=5)	123 ± 1, p<0.0001 (n=10)	127 ± 1, p<0.0001 (n=10)
LID (N=7)	172 ± 1, p<0.0001 (n=12)	132 ± 3, p<0.0001 (n=9)
LID_PREV (N=5)	65 ± 1, p<0.0001 (n=9)	142 ± 6, p=0.0029 (n=10)

Table S14: Precision measures, exact p-values, and replicate date relevant to Figure 7b.

Fig	Group1	Group 2	Mean1	Mean2	n1	n2	Sum	Adjusted p Value
Fig. 6b	LID	SHAM	128.85	99.57	10	11	***	< 0.001
	LID_CORR	SHAM	107.64	99.57	7	11	*	0.030
	LID_PREV	SHAM	96.73	99.57	11	11	ns	0.444
	LID_CORR	LID	107.64	128.85	7	10	##	0.002
	LID_PREV	LID	96.73	128.85	11	10	###	< 0.001
	LID_PREV	LID_CORR	96.73	107.64	11	7	&	0.024

Table S15: Precision measures, exact p-values, and replicate date relevant to Supplementary Figure 5

Fig	Param	Week	Group1	Group2	Mean1	Mean2	n1	n2	Sum	Adjusted p Value
Fig. Supp5	Oral	4	SHAM	SHAM_CORR	0.50	0.36	4	11	ns	1
			SHAM	SHAM_PREV	0.50	0.50	4	4	ns	1
			SHAM_CORR	SHAM_PREV	0.36	0.50	11	4	ns	1
Fig. Supp5	Oral	5	SHAM	SHAM_CORR	0.50	0.1	4	10	ns	0.51
			SHAM	SHAM_PREV	0.50	0.33	4	3	ns	0.77
			SHAM_CORR	SHAM_PREV	0.1	0.33	10	3	ns	0.60
Fig. Supp5	Oral	6	SHAM	SHAM_CORR	0.50	0.25	4	12	ns	0.49
			SHAM	SHAM_PREV	0.50	0	4	3	ns	0.49
			SHAM_CORR	SHAM_PREV	0.25	0	12	3	ns	0.49
Fig. Supp5	Oral	7	SHAM	SHAM_CORR	0.50	0	4	8	ns	0.17
			SHAM	SHAM_PREV	0.50	0.33	4	3	ns	0.77
			SHAM_CORR	SHAM_PREV	0	0.33	8	3	ns	0.18
Fig. Supp5	Oral	8	SHAM	SHAM_CORR	0.75	0.30	4	10	ns	0.33
			SHAM	SHAM_PREV	0.75	0	4	3	ns	0.33
			SHAM_CORR	SHAM_PREV	0.30	0	10	3	ns	0.33
Fig. Supp5	Oral	9	SHAM	SHAM_CORR	0.25	0.20	4	5	ns	0.94
			SHAM	SHAM_PREV	0.25	1	4	2	ns	0.33

			SHAM_CORR	SHAM_PREV	0.20	1	5	2	ns	0.33
Fig. Supp5	Axial	4	SHAM	SHAM_CORR	0	0	4	5	ns	-
			SHAM	SHAM_PREV	0	0	4	3	ns	-
			SHAM_CORR	SHAM_PREV	0	0	5	3	ns	-
Fig. Supp5	Axial	5	SHAM	SHAM_CORR	0	0	4	3	ns	-
			SHAM	SHAM_PREV	0	0	4	2	ns	-
			SHAM_CORR	SHAM_PREV	0	0	3	2	ns	-
Fig. Supp5	Axial	6	SHAM	SHAM_CORR	0	0	4	5	ns	-
			SHAM	SHAM_PREV	0	0	4	2	ns	-
			SHAM_CORR	SHAM_PREV	0	0	5	2	ns	-
Fig. Supp5	Axial	7	SHAM	SHAM_CORR	0	0	4	5	ns	-
			SHAM	SHAM_PREV	0	0	4	2	ns	-
			SHAM_CORR	SHAM_PREV	0	0	5	2	ns	-
Fig. Supp5	Axial	8	SHAM	SHAM_CORR	0	0	4	5	ns	-
			SHAM	SHAM_PREV	0	0	4	2	ns	-
			SHAM_CORR	SHAM_PREV	0	0	5	2	ns	-
Fig. Supp5	Axial	9	SHAM	SHAM_CORR	0	0	4	5	ns	-
			SHAM	SHAM_PREV	0	0	4	2	ns	-
			SHAM_CORR	SHAM_PREV	0	0	5	2	ns	-
Fig.	Limb	4	SHAM	SHAM_CORR	0	0.14	4	7	ns	0.51

Supp5			SHAM	SHAM_PREV	0	0	4	3	ns	-
			SHAM_CORR	SHAM_PREV	0.14	0	7	3	ns	0.51
Fig. Supp5	Limb	5	SHAM	SHAM_CORR	0	0.20	4	5	ns	0.54
			SHAM	SHAM_PREV	0	0	4	2	ns	-
			SHAM_CORR	SHAM_PREV	0.20	0	5	2	ns	0.54
Fig. Supp5	Limb	6	SHAM	SHAM_CORR	0	0	4	7	ns	-
			SHAM	SHAM_PREV	0	0	4	2	ns	-
			SHAM_CORR	SHAM_PREV	0	0	7	2	ns	-
Fig. Supp5	Limb	7	SHAM	SHAM_CORR	0	0.17	4	6	ns	0.58
			SHAM	SHAM_PREV	0	0	4	2	ns	-
			SHAM_CORR	SHAM_PREV	0.17	0	6	2	ns	0.58
Fig. Supp5	Limb	8	SHAM	SHAM_CORR	0	0.17	4	6	ns	0.58
			SHAM	SHAM_PREV	0	0	4	2	ns	-
			SHAM_CORR	SHAM_PREV	0.17	0	6	2	ns	0.58
Fig. Supp5	Limb	9	SHAM	SHAM_CORR	0	0.17	4	6	ns	0.58
			SHAM	SHAM_PREV	0	0	4	2	ns	-
			SHAM_CORR	SHAM_PREV	0.17	0	6	2	ns	0.58

Table S16: Precision measures, exact p-values, and replicate date relevant to Supplementary Figure 8

Fig	Reg	Group	Weeks1	Weeks2	Mean1	Mean2	n1	n2	Sum	Adjusted p Value
Fig. Supp8 (upper)	IN	SHAM	W2-W3	W4-W5	2.64 Hz	2.52 Hz	10	11	ns	0.975
			W2-W3	W6-W7	2.64 Hz	2.12 Hz	10	10	ns	0.551
			W2-W3	W8-W9	2.64 Hz	2.88 Hz	10	9	ns	0.854
			W4-W5	W6-W7	2.52 Hz	2.12 Hz	11	10	ns	0.743
			W4-W5	W8-W9	2.52 Hz	2.88 Hz	11	9	ns	0.617
			W6-W7	W8-W9	2.12 Hz	2.88 Hz	10	9	ns	0.223
Fig. Supp8 (upper)	IN	SHAM CORR	W2-W3	W4-W5	2.37 Hz	1.89 Hz	13	21	ns	0.228
			W2-W3	W6-W7	2.37 Hz	2.27 Hz	13	21	***	<0.001
			W2-W3	W8-W9	2.37 Hz	2.28 Hz	13	24	ns	0.472
			W4-W5	W6-W7	1.89 Hz	2.27 Hz	21	21	*	0.018
			W4-W5	W8-W9	1.89 Hz	2.28 Hz	21	24	ns	0.914
			W6-W7	W8-W9	2.27 Hz	2.28 Hz	21	24	**	0.002
Fig. Supp8 (upper)	IN	SHAM PREV	W2-W3	W4-W5	2.84 Hz	1.92 Hz	18	23	***	< 0.001
			W2-W3	W6-W7	2.84 Hz	2.14 Hz	18	19	***	< 0.001
			W2-W3	W8-W9	2.84 Hz	2.32 Hz	18	16	*	0.016
			W4-W5	W6-W7	1.92 Hz	2.14 Hz	23	19	ns	0.492
			W4-W5	W8-W9	1.92 Hz	2.32 Hz	23	16	ns	0.076
			W6-W7	W8-W9	2.14 Hz	2.32 Hz	19	16	ns	0.716
Fig.	DN	SHAM	W2-W3	W4-W5	2.82 Hz	2.66 Hz	28	30	ns	0.406
			W2-W3	W6-W7	2.82 Hz	2.60 Hz	28	24	ns	0.742

Supp8 (upper)			W2-W3	W8-W9	2.82 Hz	2.73 Hz	28	28	ns	0.806
			W4-W5	W6-W7	2.66 Hz	2.60 Hz	30	24	ns	0.998
			W4-W5	W8-W9	2.66 Hz	2.73 Hz	30	28	ns	0.916
			W6-W7	W8-W9	2.60 Hz	2.73 Hz	24	28	ns	0.954
Fig. Supp8 (upper)	DN	SHAM PREV	W2-W3	W4-W5	3.14 Hz	2.15 Hz	10	12	***	< 0.001
			W2-W3	W6-W7	3.14 Hz	2.08 Hz	10	11	***	< 0.001
			W2-W3	W8-W9	3.14 Hz	2.57 Hz	10	13	*	0.024
			W4-W5	W6-W7	2.15 Hz	2.08 Hz	12	11	ns	0.982
			W4-W5	W8-W9	2.15 Hz	2.57 Hz	12	13	ns	0.126
			W6-W7	W8-W9	2.08 Hz	2.57 Hz	11	13	ns	0.062
Fig. Supp8 (upper)	FN	SHAM	W2-W3	W4-W5	2.71 Hz	2.69 Hz	7	8	ns	1
			W2-W3	W6-W7	2.71 Hz	1.99 Hz	7	3	ns	0.191
			W2-W3	W8-W9	2.71 Hz	2.40 Hz	7	3	ns	0.808
			W4-W5	W6-W7	2.69 Hz	1.99 Hz	8	3	ns	0.193
			W4-W5	W8-W9	2.69 Hz	2.40 Hz	8	3	ns	0.821
			W6-W7	W8-W9	1.99 Hz	2.40 Hz	3	3	ns	0.739
Fig. Supp8 (upper)	FN	SHAM PREV	W2-W3	W4-W5	3.25 Hz	2.34 Hz	18	14	***	<0.001
			W2-W3	W6-W7	3.25 Hz	1.99 Hz	18	15	***	<0.001
			W2-W3	W8-W9	3.25 Hz	2.28 Hz	18	16	***	<0.001
			W4-W5	W6-W7	2.34 Hz	1.99 Hz	14	15	ns	0.290
			W4-W5	W8-W9	2.34 Hz	2.28 Hz	14	16	ns	0.990
			W6-W7	W8-W9	1.99 Hz	2.28 Hz	15	16	ns	0.423

Fig. Supp8 (middle)	IN	SHAM	W2-W3	W4-W5	28.58 Hz	22.26 Hz	10	11	ns	0.582
			W2-W3	W6-W7	28.58 Hz	12.35 Hz	10	5	*	0.047
			W2-W3	W8-W9	28.58 Hz	27.84 Hz	10	9	ns	0.999
			W4-W5	W6-W7	22.26 Hz	12.35 Hz	11	5	ns	0.104
			W4-W5	W8-W9	22.26 Hz	27.84 Hz	11	9	ns	0.725
			W6-W7	W8-W9	12.35 Hz	27.84 Hz	5	9	ns	0.087
Fig. Supp8 (middle)	IN	SHAM CORR	W2-W3	W4-W5	38.92 Hz	14.03 Hz	7	9	***	<0.001
			W2-W3	W6-W7	38.92 Hz	24.58 Hz	7	9	**	0.008
			W2-W3	W8-W9	38.92 Hz	26.18 Hz	7	12	*	0.013
			W4-W5	W6-W7	14.03 Hz	24.58 Hz	9	9	*	0.048
			W4-W5	W8-W9	14.03 Hz	26.18 Hz	9	12	*	0.010
			W6-W7	W8-W9	24.58 Hz	26.18 Hz	9	12	ns	0.970
Fig. Supp8 (middle)	IN	SHAM PREV	W2-W3	W4-W5	28.28 Hz	18.96 Hz	18	23	**	0.001
			W2-W3	W6-W7	28.28 Hz	19.39 Hz	18	19	**	0.004
			W2-W3	W8-W9	28.28 Hz	21.53 Hz	18	16	ns	0.060
			W4-W5	W6-W7	18.96 Hz	19.39 Hz	23	19	ns	0.998
			W4-W5	W8-W9	18.96 Hz	21.53 Hz	23	16	ns	0.735
			W6-W7	W8-W9	19.39 Hz	21.53 Hz	19	16	ns	0.846
Fig. Supp8 (middle)	DN	SHAM	W2-W3	W4-W5	29.96 Hz	22.64 Hz	28	30	ns	0.191
			W2-W3	W6-W7	29.96 Hz	19.02 Hz	28	5	ns	0.369
			W2-W3	W8-W9	29.96 Hz	26.47 Hz	28	28	ns	0.782
			W4-W5	W6-W7	22.64 Hz	19.02 Hz	30	5	ns	0.949

			W4-W5	W8-W9	22.64 Hz	26.47 Hz	30	28	ns	0.718
			W6-W7	W8-W9	19.02 Hz	26.47 Hz	5	28	ns	0.685
Fig. Supp8 (middle)	DN	SHAM PREV	W2-W3	W4-W5	29.52 Hz	18.95 Hz	10	12	**	0.006
			W2-W3	W6-W7	29.52 Hz	21.48 Hz	10	11	ns	0.060
			W2-W3	W8-W9	29.52 Hz	19.48 Hz	10	13	**	0.008
			W4-W5	W6-W7	18.95 Hz	21.48 Hz	12	11	ns	0.828
			W4-W5	W8-W9	18.95 Hz	19.48 Hz	12	13	ns	0.997
			W6-W7	W8-W9	21.48 Hz	19.48 Hz	11	13	ns	0.902
Fig. Supp8 (middle)	FN	SHAM	W2-W3	W4-W5	24.46 Hz	25.18 Hz	7	8	ns	0.988
			W2-W3	W8-W9	24.46 Hz	19.50 Hz	7	3	ns	0.737
			W4-W5	W8-W9	25.18 Hz	19.50 Hz	8	3	ns	0.662
Fig. Supp8 (middle)	FN	SHAM PREV	W2-W3	W4-W5	34.79 Hz	15.57 Hz	18	14	***	<0.001
			W2-W3	W6-W7	34.79 Hz	12.11 Hz	18	15	***	<0.001
			W2-W3	W8-W9	34.79 Hz	18.13 Hz	18	16	***	<0.001
			W4-W5	W6-W7	15.57 Hz	12.11 Hz	14	15	ns	0.774
			W4-W5	W8-W9	15.57 Hz	18.13 Hz	14	16	ns	0.888
			W6-W7	W8-W9	12.11 Hz	18.13 Hz	15	16	ns	0.320
Fig. Supp8 (lower)	IN	SHAM	W2-W3	W4-W5	18.12 Hz	13.31 Hz	10	11	ns	0.749
			W2-W3	W6-W7	18.12 Hz	7.41 Hz	10	5	ns	0.303
			W2-W3	W8-W9	18.12 Hz	18.49 Hz	10	9	ns	1
			W4-W5	W6-W7	13.31 Hz	7.41 Hz	11	5	ns	0.754
			W4-W5	W8-W9	13.31 Hz	18.49 Hz	11	9	ns	0.723

			W6-W7	W8-W9	7.41 Hz	18.49 Hz	5	9	ns	0.290
Fig. Supp8 (lower)	IN	SHAM CORR	W2-W3	W4-W5	11.99 Hz	10.13 Hz	12	9	ns	0.561
			W2-W3	W6-W7	11.99 Hz	6.01 Hz	12	9	***	< 0.001
			W2-W3	W8-W9	11.99 Hz	10.57 Hz	12	12	ns	0.703
			W4-W5	W6-W7	10.13 Hz	6.01 Hz	9	9	*	0.047
			W4-W5	W8-W9	10.13 Hz	10.57 Hz	9	12	ns	0.989
			W6-W7	W8-W9	6.01 Hz	10.57 Hz	9	12	*	0.013
Fig. Supp8 (lower)	IN	SHAM PREV	W2-W3	W4-W5	15.70 Hz	6.49 Hz	18	23	***	<0.001
			W2-W3	W6-W7	15.70 Hz	8.38 Hz	18	19	**	0.002
			W2-W3	W8-W9	15.70 Hz	10.02 Hz	18	16	*	0.036
			W4-W5	W6-W7	6.49 Hz	8.38 Hz	23	19	ns	0.307
			W4-W5	W8-W9	6.49 Hz	10.02 Hz	23	16	ns	0.074
			W6-W7	W8-W9	8.38 Hz	10.02 Hz	19	16	ns	0.703
Fig. Supp8 (lower)	DN	SHAM	W2-W3	W4-W5	15.57 Hz	15.49 Hz	28	30	ns	1
			W2-W3	W6-W7	15.57 Hz	14.47 Hz	28	19	ns	0.980
			W2-W3	W8-W9	15.57 Hz	15.62 Hz	28	28	ns	1
			W4-W5	W6-W7	15.49 Hz	14.47 Hz	30	19	ns	0.984
			W4-W5	W8-W9	15.49 Hz	15.62 Hz	30	28	ns	1
			W6-W7	W8-W9	14.47 Hz	15.62 Hz	19	28	ns	0.977
Fig. Supp8 (lower)	DN	SHAM PREV	W2-W3	W4-W5	25.75 Hz	8.78 Hz	10	12	***	<0.001
			W2-W3	W6-W7	25.75 Hz	8.23 Hz	10	11	***	<0.001
			W2-W3	W8-W9	25.75 Hz	12.22 Hz	10	13	***	<0.001

			W4-W5	W6-W7	8.78 Hz	8.23 Hz	12	11	ns	0.997
			W4-W5	W8-W9	8.78 Hz	12.22 Hz	12	13	ns	0.570
			W6-W7	W8-W9	8.23 Hz	12.22 Hz	11	13	ns	0.464
Fig. Supp8 (lower)	FN	SHAM	W2-W3	W4-W5	15.59 Hz	15.16 Hz	7	8	ns	0.994
			W2-W3	W8-W9	15.59 Hz	10.37 Hz	7	3	ns	0.623
			W4-W5	W8-W9	15.16 Hz	10.37 Hz	8	3	ns	0.660
Fig. Supp8 (lower)	FN	SHAM PREV	W2-W3	W4-W5	26.22 Hz	11.01 Hz	18	14	***	<0.001
			W2-W3	W6-W7	26.22 Hz	7.32 Hz	18	15	***	<0.001
			W2-W3	W8-W9	26.22 Hz	11.81 Hz	18	16	***	<0.001
			W4-W5	W6-W7	11.01 Hz	7.32 Hz	14	15	ns	0.714
			W4-W5	W8-W9	11.01 Hz	11.81 Hz	14	16	ns	0.995
			W6-W7	W8-W9	7.32 Hz	11.81 Hz	15	16	ns	0.545

Table S17: Precision measures, exact p-values, and replicate date relevant to Supplementary Figure9a

**Fig**	**Reg**	**Group**	**Weeks1**	**Weeks2**	**Mean1**	**Mean2**	**n1**	**n2**	**Sum**	**Adjusted p Value**
Fig. Supp9a	IN	SHAM	W2-W3	W4-W5	1.78 Hz	2.25 Hz	10	11	ns	0.768
			W2-W3	W6-W7	1.78 Hz	0.30 Hz	10	5	ns	0.091
			W2-W3	W8-W9	1.78 Hz	2.20 Hz	10	9	ns	0.839
			W4-W5	W6-W7	2.25 Hz	0.30 Hz	11	5	*	0.014
			W4-W5	W8-W9	2.25 Hz	2.20 Hz	11	9	ns	1

			W6-W7	W8-W9	0.30 Hz	2.20 Hz	5	9	*	0.021
Fig. Supp9a	IN	LID	W2-W3	W4-W5	1.25 Hz	0.26 Hz	25	8	*	0.011
			W2-W3	W6-W7	1.25 Hz	0.12 Hz	25	9	**	0.002
			W2-W3	W8-W9	1.25 Hz	0.22 Hz	25	13	**	0.006
			W4-W5	W6-W7	0.26 Hz	0.12 Hz	8	9	ns	0.400
			W4-W5	W8-W9	0.26 Hz	0.22 Hz	8	13	ns	0.949
			W6-W7	W8-W9	0.12 Hz	0.22 Hz	9	13	ns	0.053
Fig. Supp9a	IN	LID CORR	W2-W3	W4-W5	0.70 Hz	2.70 Hz	19	24	ns	0.193
			W2-W3	W6-W7	0.70 Hz	0.94 Hz	19	12	ns	0.997
			W2-W3	W8-W9	0.70 Hz	2.10 Hz	19	11	ns	0.664
			W4-W5	W6-W7	2.70 Hz	0.94 Hz	24	12	ns	0.418
			W4-W5	W8-W9	2.70 Hz	2.10 Hz	24	11	ns	0.956
			W6-W7	W8-W9	0.94 Hz	2.10 Hz	12	11	ns	0.825
Fig. Supp9a	IN	LID PREV	W2-W3	W4-W5	0.94 Hz	1.03 Hz	31	32	ns	0.979
			W2-W3	W6-W7	0.94 Hz	1.34 Hz	31	26	ns	0.407
			W2-W3	W8-W9	0.94 Hz	1.31 Hz	31	27	ns	0.477
			W4-W5	W6-W7	1.03 Hz	1.34 Hz	32	26	ns	0.633
			W4-W5	W8-W9	1.03 Hz	1.31 Hz	32	27	ns	0.708
			W6-W7	W8-W9	1.34 Hz	1.31 Hz	26	27	ns	0.999
Fig. Supp9a	DN	SHAM	W2-W3	W4-W5	1.21 Hz	1.52 Hz	28	30	ns	0.853
			W2-W3	W6-W7	1.21 Hz	0.60 Hz	28	5	ns	0.825
			W2-W3	W8-W9	1.21 Hz	1.74 Hz	28	28	ns	0.538

			W4-W5	W6-W7	1.52 Hz	0.60 Hz	30	5	ns	0.564
			W4-W5	W8-W9	1.52 Hz	1.74 Hz	30	28	ns	0.942
			W6-W7	W8-W9	0.60 Hz	1.74 Hz	5	28	ns	0.383
Fig. Supp9a	DN	LID	W2-W3	W4-W5	1.27 Hz	0.25 Hz	41	33	***	<0.001
			W2-W3	W6-W7	1.27 Hz	0.40 Hz	41	17	**	0.002
			W2-W3	W8-W9	1.27 Hz	0.28 Hz	41	27	***	<0.001
			W4-W5	W6-W7	0.25 Hz	0.40 Hz	33	17	ns	0.447
			W4-W5	W8-W9	0.25 Hz	0.28 Hz	33	27	ns	0.984
			W6-W7	W8-W9	0.40 Hz	0.28 Hz	17	27	ns	0.702
Fig. Supp9a	DN	LID CORR	W2-W3	W4-W5	1.56 Hz	0.27 Hz	12	17	ns	0.083
			W2-W3	W6-W7	1.56 Hz	0.36 Hz	12	10	ns	0.115
			W2-W3	W8-W9	1.56 Hz	0.38 Hz	12	12	ns	0.122
			W4-W5	W6-W7	0.27 Hz	0.36 Hz	17	10	ns	0.814
			W4-W5	W8-W9	0.27 Hz	0.38 Hz	17	12	ns	0.641
			W6-W7	W8-W9	0.36 Hz	0.38 Hz	10	12	ns	0.998
Fig. Supp9a	DN	LID PREV	W2-W3	W4-W5	1.77 Hz	1.11 Hz	34	26	ns	0.339
			W2-W3	W6-W7	1.77 Hz	0.52 Hz	34	25	*	0.010
			W2-W3	W8-W9	1.77 Hz	1.04 Hz	34	16	ns	0.372
			W4-W5	W6-W7	1.11 Hz	0.52 Hz	26	25	ns	0.481
			W4-W5	W8-W9	1.11 Hz	1.04 Hz	26	16	ns	0.998
			W6-W7	W8-W9	0.52 Hz	1.04 Hz	25	16	ns	0.698
Fig.	FN	SHAM	W2-W3	W4-W5	1.56 Hz	2.71 Hz	7	8	ns	0.175

Supp9a			W2-W3	W8-W9	1.56 Hz	2.01 Hz	7	3	ns	0.845
			W4-W5	W8-W9	2.71 Hz	2.01 Hz	8	3	ns	0.661
Fig. Supp9a	FN	LID	W2-W3	W4-W5	0.92 Hz	0.65 Hz	41	42	ns	0.592
			W2-W3	W6-W7	0.92 Hz	0.32 Hz	41	49	*	0.015
			W2-W3	W8-W9	0.92 Hz	0.44 Hz	41	27	ns	0.080
			W4-W5	W6-W7	0.65 Hz	0.32 Hz	42	49	*	0.025
			W4-W5	W8-W9	0.65 Hz	0.44 Hz	42	27	ns	0.310
			W6-W7	W8-W9	0.32 Hz	0.44 Hz	49	27	ns	0.188
Fig. Supp9a	FN	LID CORR	W2-W3	W4-W5	1.22 Hz	0.95 Hz	15	22	ns	0.940
			W2-W3	W6-W7	1.22 Hz	0.56 Hz	15	20	ns	0.505
			W2-W3	W8-W9	1.22 Hz	1.20 Hz	15	17	ns	1
			W4-W5	W6-W7	0.95 Hz	0.56 Hz	22	20	ns	0.791
			W4-W5	W8-W9	0.95 Hz	1.20 Hz	22	17	ns	0.943
			W6-W7	W8-W9	0.56 Hz	1.20 Hz	20	17	ns	0.494
Fig. Supp9a	FN	LID PREV	W2-W3	W4-W5	2.27 Hz	1.09 Hz	31	32	ns	0.120
			W2-W3	W6-W7	2.27 Hz	0.68 Hz	31	32	**	0.001
			W2-W3	W8-W9	2.27 Hz	0.86 Hz	31	29	*	0.031
			W4-W5	W6-W7	1.09 Hz	0.68 Hz	32	32	ns	0.341
			W4-W5	W8-W9	1.09 Hz	0.86 Hz	32	29	ns	0.833
			W6-W7	W8-W9	0.68 Hz	0.86 Hz	32	29	ns	0.751

Table S18: Precision measures, exact p-values, and replicate date relevant to Supplementary Figure9b

Fig	Reg	Group	Weeks1	Weeks2	Mean1	Mean2	n1	n2	Sum	Adjusted p Value
Fig. Supp9b	IN	SHAM	W2-W3	W4-W5	1.19 Hz	0.58 Hz	10	11	ns	0.504
			W2-W3	W6-W7	1.19 Hz	0.19 Hz	10	5	ns	0.141
			W2-W3	W8-W9	1.19 Hz	0.30 Hz	10	9	ns	0.211
			W4-W5	W6-W7	0.58 Hz	0.19 Hz	11	5	**	0.005
			W4-W5	W8-W9	0.58 Hz	0.30 Hz	11	9	ns	0.050
			W6-W7	W8-W9	0.19 Hz	0.30 Hz	5	9	ns	0.173
Fig. Supp9b	IN	LID	W2-W3	W4-W5	0.77 Hz	1.39 Hz	25	8	ns	0.773
			W2-W3	W6-W7	0.77 Hz	1.01 Hz	25	9	ns	0.981
			W2-W3	W8-W9	0.77 Hz	1.53 Hz	25	13	ns	0.507
			W4-W5	W6-W7	1.39 Hz	1.01 Hz	8	9	ns	0.960
			W4-W5	W8-W9	1.39 Hz	1.53 Hz	8	13	ns	0.997
			W6-W7	W8-W9	1.01 Hz	1.53 Hz	9	13	ns	0.873
Fig. Supp9b	IN	LID CORR	W2-W3	W4-W5	0.49 Hz	0.56 Hz	19	24	ns	0.974
			W2-W3	W6-W7	0.49 Hz	1.10 Hz	19	12	**	0.005
			W2-W3	W8-W9	0.49 Hz	0.60 Hz	19	11	ns	0.926
			W4-W5	W6-W7	0.56 Hz	1.10 Hz	24	12	**	0.009
			W4-W5	W8-W9	0.56 Hz	0.60 Hz	24	11	ns	0.992
			W6-W7	W8-W9	1.10 Hz	0.60 Hz	12	11	ns	0.067

Fig. Supp9b	IN	LID PREV	W2-W3	W4-W5	0.78 Hz	0.94 Hz	31	32	ns	0.641
			W2-W3	W6-W7	0.78 Hz	1.05 Hz	31	26	ns	0.126
			W2-W3	W8-W9	0.78 Hz	0.73 Hz	31	27	ns	0.976
			W4-W5	W6-W7	0.94 Hz	1.05 Hz	32	26	ns	0.871
			W4-W5	W8-W9	0.94 Hz	0.73 Hz	32	27	ns	0.421
			W6-W7	W8-W9	1.05 Hz	0.73 Hz	26	27	ns	0.051
Fig. Supp9b	DN	SHAM	W2-W3	W4-W5	0.46 Hz	0.39 Hz	28	30	ns	0.884
			W2-W3	W6-W7	0.46 Hz	0.31 Hz	28	5	ns	0.862
			W2-W3	W8-W9	0.46 Hz	0.48 Hz	28	28	ns	0.996
			W4-W5	W6-W7	0.39 Hz	0.31 Hz	30	5	ns	0.980
			W4-W5	W8-W9	0.39 Hz	0.48 Hz	30	28	ns	0.778
			W6-W7	W8-W9	0.31 Hz	0.48 Hz	5	28	ns	0.805
Fig. Supp9b	DN	LID	W2-W3	W4-W5	0.61 Hz	1.19 Hz	41	33	***	<0.001
			W2-W3	W6-W7	0.61 Hz	2.47 Hz	41	17	ns	0.299
			W2-W3	W8-W9	0.61 Hz	3.10 Hz	41	27	***	<0.001
			W4-W5	W6-W7	1.19 Hz	2.47 Hz	33	17	ns	0.609
			W4-W5	W8-W9	1.19 Hz	3.10 Hz	33	27	**	0.006
			W6-W7	W8-W9	2.47 Hz	3.10 Hz	17	27	ns	0.943
Fig. Supp9b	DN	LID CORR	W2-W3	W4-W5	0.42 Hz	0.66 Hz	12	17	ns	0.371
			W2-W3	W6-W7	0.42 Hz	0.44 Hz	12	10	ns	0.999
			W2-W3	W8-W9	0.42 Hz	0.42 Hz	12	12	ns	1
			W4-W5	W6-W7	0.66 Hz	0.44 Hz	17	10	ns	0.495

			W4-W5	W8-W9	0.66 Hz	0.42 Hz	17	12	ns	0.363
			W6-W7	W8-W9	0.44 Hz	0.42 Hz	10	12	ns	0.999
Fig. Supp9b	DN	LID PREV	W2-W3	W4-W5	0.47 Hz	0.86 Hz	34	26	**	0.009
			W2-W3	W6-W7	0.47 Hz	1.52 Hz	34	25	***	<0.001
			W2-W3	W8-W9	0.47 Hz	0.60 Hz	34	16	ns	0.324
			W4-W5	W6-W7	0.86 Hz	1.52 Hz	26	25	ns	0.061
			W4-W5	W8-W9	0.86 Hz	0.60 Hz	26	16	ns	0.192
			W6-W7	W8-W9	1.52 Hz	0.60 Hz	25	16	**	0.004
Fig. Supp9b	FN	SHAM	W2-W3	W4-W5	0.85 Hz	0.58 Hz	7	8	ns	0.383
			W2-W3	W8-W9	0.85 Hz	0.25 Hz	7	3	ns	0.083
			W4-W5	W8-W9	0.58 Hz	0.25 Hz	8	3	ns	0.403
Fig. Supp9b	FN	LID	W2-W3	W4-W5	0.64 Hz	1.00 Hz	41	42	ns	0.435
			W2-W3	W6-W7	0.64 Hz	2.91 Hz	41	49	**	0.006
			W2-W3	W8-W9	0.64 Hz	1.27 Hz	41	27	ns	0.191
			W4-W5	W6-W7	1.00 Hz	2.91 Hz	42	49	*	0.019
			W4-W5	W8-W9	1.00 Hz	1.27 Hz	42	27	ns	0.670
			W6-W7	W8-W9	2.91 Hz	1.27 Hz	49	27	ns	0.073
Fig. Supp9b	FN	LID CORR	W2-W3	W4-W5	1.65 Hz	0.73 Hz	15	22	ns	0.500
			W2-W3	W6-W7	1.65 Hz	0.79 Hz	15	20	ns	0.559
			W2-W3	W8-W9	1.65 Hz	0.66 Hz	15	17	ns	0.448
			W4-W5	W6-W7	0.73 Hz	0.79 Hz	22	20	ns	0.932
			W4-W5	W8-W9	0.73 Hz	0.66 Hz	22	17	ns	0.948

			W6-W7	W8-W9	0.79 Hz	0.66 Hz	20	17	ns	0.685
Fig. Supp9b	FN	LID PREV	W2-W3	W4-W5	0.71 Hz	0.99 Hz	31	32	ns	0.072
			W2-W3	W6-W7	0.71 Hz	0.73 Hz	31	32	ns	0.993
			W2-W3	W8-W9	0.71 Hz	0.54 Hz	31	29	ns	0.136
			W4-W5	W6-W7	0.99 Hz	0.73 Hz	32	32	ns	0.134
			W4-W5	W8-W9	0.99 Hz	0.54 Hz	32	29	***	<0.001
			W6-W7	W8-W9	0.73 Hz	0.54 Hz	32	29	ns	0.105

SUPPLEMENTARY TEXT

Influence of locomotion on CN discharge

As CN activity is modulated by locomotion in normal animals[1], we quantified locomotor activity using DeepLabCut [2] (**Figure S7**) and tested if the activity observed in CN correlated with a change in locomotor activity in SHAM animals (**Figure S8**). No differences were observed in the CN during levodopa treatment in SHAM animals, whether the animals were moving or not (**Supplementary Figure 8**).

Increased cv2.isi does not simply reflect increased bursting

Since greater cv2.isi in LID mice may reflect irregular burst firing under levodopa treatment in CN neurons, we analyzed the burst rate in periods of locomotor activity and inactivity. Surprisingly, the burst rate of dyskinetic mice decreased during levodopa treatment in periods of activity in the IN, DN, and FN (**Figure S9a**). However, the burst rate of LID mice in periods of locomotor inactivity increased during levodopa treatment in DN and FN (**Figure S9b**).

PC stimulation during 4 weeks prevented the decreased of the burst rate in IN during periods of locomotor activity (**Figure S9a, left panel**). Changes, although less consistent, were also observed as a function of the motor state in DN and FN (**Figure S9a, middle and right panels)**. Only mild differences were observed in the burst rate in IN, DN or FN in the different groups during periods of locomotor inactivity (**Figure S9b)**.

REFERENCES

1. Sarnaik R, Raman IM. Control of voluntary and optogenetically perturbed locomotion by spike rate and timing of neurons of the mouse cerebellar nuclei. *Elife* **7**, (2018).

2. Mathis A*, et al.* DeepLabCut: markerless pose estimation of user-defined body parts with deep learning. *Nat Neurosci* **21**, 1281-1289 (2018).
