# Supplementary material for: Cerebellar stimulation prevents Levodopa-induced dyskinesia in mice and normalizes activity in a motor network
Source: Nat Commun. 2022 Jun 9;13:3211. doi: 10.1038/s41467-022-30844-0 (PMC9184492; doi:10.1038/s41467-022-30844-0)
Supplement: Supplementary file 2 — Reporting Summary [file 41467_2022_30844_MOESM2_ESM.pdf]

nature portfolio

Corresponding author(s): Daniela Popa, Clément Léna

Last updated by author(s): May 9th, 2022

Reporting Summary

Nature Portfolio wishes to improve the reproducibility of the work that we publish. This form provides structure for consistency and transparency in reporting. For further information on Nature Portfolio policies, see our Editorial Policies and the Editorial Policy Checklist.

Statistics

For all statistical analyses, confirm that the following items are present in the figure legend, table legend, main text, or Methods section.

n/a	Confirmed	
☐	☒	The exact sample size (*n*) for each experimental group/condition, given as a discrete number and unit of measurement
☐	☒	A statement on whether measurements were taken from distinct samples or whether the same sample was measured repeatedly
☐	☒	The statistical test(s) used AND whether they are one- or two-sided<br>*Only common tests should be described solely by name; describe more complex techniques in the Methods section.*
☒	☐	A description of all covariates tested
☐	☒	A description of any assumptions or corrections, such as tests of normality and adjustment for multiple comparisons
☐	☒	A full description of the statistical parameters including central tendency (e.g. means) or other basic estimates (e.g. regression coefficient) AND variation (e.g. standard deviation) or associated estimates of uncertainty (e.g. confidence intervals)
☐	☒	For null hypothesis testing, the test statistic (e.g. *F*, *t*, *r*) with confidence intervals, effect sizes, degrees of freedom and *P* value noted<br>*Give P values as exact values whenever suitable.*
☒	☐	For Bayesian analysis, information on the choice of priors and Markov chain Monte Carlo settings
☒	☐	For hierarchical and complex designs, identification of the appropriate level for tests and full reporting of outcomes
☒	☐	Estimates of effect sizes (e.g. Cohen's *d*, Pearson's *r*), indicating how they were calculated

Our web collection on statistics for biologists contains articles on many of the points above.

Software and code

Policy information about availability of computer code

Data collection	Data collection were performed using multichannel Tucker Davis Technology's recording system with their Synapse Software.
Data analysis	Matlab R2006a, R Studio software 4.1.2, DeepLabCut, Prism 7, ImageJ-win64 v1.53q

For manuscripts utilizing custom algorithms or software that are central to the research but not yet described in published literature, software must be made available to editors and reviewers. We strongly encourage code deposition in a community repository (e.g. GitHub). See the Nature Portfolio guidelines for submitting code & software for further information.

Data

Policy information about availability of data

All manuscripts must include a data availability statement. This statement should provide the following information, where applicable:

- Accession codes, unique identifiers, or web links for publicly available datasets
- A description of any restrictions on data availability
- For clinical datasets or third party data, please ensure that the statement adheres to our policy

All data that are available in the main text or in the supplementary materials and that support the findings of this study (Fig. 1-8, Supplementary Figures 1-10) are available from the corresponding author upon reasonable request. Source data are provided as a Source Data file with this paper. Additional file containing the datasets to verify, interpret, reproduce, and analyze all figures and supplementary figures in the study as well as the related codes will be provided with the paper and on GitHub. For additional information, please refer to the corresponding Life Sciences Reporting Summary.

Field-specific reporting

Please select the one below that is the best fit for your research. If you are not sure, read the appropriate sections before making your selection.

☒ Life sciences ☐ Behavioural & social sciences ☐ Ecological, evolutionary & environmental sciences

For a reference copy of the document with all sections, see nature.com/documents/nr-reporting-summary-flat.pdf

Life sciences study design

All studies must disclose on these points even when the disclosure is negative.

Sample size	Our sample-size are consistent with sample size's in the literature (e.g. Calderon et al Nat Neurosci 2011, Calabresi et al. Nat Neurosci 2016, Cenci et al Neuroscience 1999, Valverde et al Nat Commun 2020, Miterko et al Nat Commun 2021). Sample size was determined to be adequate based on the magnitude and consistency of measurable differences between groups. The sample size (n) of each experiment is provided in the corresponding figure captions in the main manuscript and in materials and methods section. Sample sizes were chosen to support meaningful conclusions in accordance with ethical committee requirements to limit as much as possible the use of animals.
Data exclusions	If postmortem studies showed evidence of inadequate dopamine depletion, ChR2 expression, electrode's implantation, or off-target expression of viruses, mice were excluded from further analysis.
Replication	Each experiment presented in this paper was repeated in multiple series of animals (3-5 times). Each series contained animals assigned to different groups. The observations from each group thus derive from multiple independent experiments. All attempts of replications of individual experiment were successfull in terms of similar outcomes.
Randomization	Animals were assigned randomly to experimental and control groups.
Blinding	Unless otherwise stated, all experiments were performed several times and by several experimenters, blinded to the experimental groups. The data collection for the in vivo electrophysiological and chemogenetic experiments were not performed by experimenters blinded to the condition as the different conditions required different treatments. However, for the data analysis of these experiments, experimenters were blinded to the experimental groups.

Reporting for specific materials, systems and methods

We require information from authors about some types of materials, experimental systems and methods used in many studies. Here, indicate whether each material, system or method listed is relevant to your study. If you are not sure if a list item applies to your research, read the appropriate section before selecting a response.

Materials & experimental systems

n/a	Involved in the study	
☐	☒	Antibodies
☒	☐	Eukaryotic cell lines
☒	☐	Palaeontology and archaeology
☐	☒	Animals and other organisms
☒	☐	Human research participants
☒	☐	Clinical data
☒	☐	Dual use research of concern

Methods

n/a	Involved in the study	
☒	☐	ChIP-seq
☒	☐	Flow cytometry
☒	☐	MRI-based neuroimaging

Antibodies

Antibodies used	Anti-Tyrosine hydroxylase #2 Polyclonal Guinea Pig Antibody (Synaptic System, Cat.No. 213 104, 1:500);<br>Sc-48 Rabbit anti-FosB (Santa Cruz Biotechnology, Sc-398595, 1:100)<br>Cy3-AffiniPure Donkey anti-Guinea Pig IgG (H+L) (Jackson ImmunoResearch Labs, 706-165-148, 1:400);<br>Alexa Fluor 488-AffiniPure Goat anti-Rabbit IgG (H+L) (Jackson ImmunoResearch Labs, 111-545-144, 1:200);<br>Hoechst 33342 Trihydrochloride trihydrate (Life Technologies SAS, H3570, 1:10 000)
Validation	Anti-Tyrosine hydroxylase #2 Polyclonal Guinea Pig Antibody (Synaptic System, Cat.No. 213 104, 1:500); Reacts with: rat (P04177), mouse (P24529). Other species not tested yet. Validated use for WB: 1 : 1000 (AP staining), IP, IHC: 1 : 500, IHC-P/FFPE: 1 : 500 for specific detection of tyrosine hydroxylase without cross-reactivity to tryptophane hydroxylase. References: Hartlage-Rübsamen M, et al. Acta neuropathologica (2021) - IHC; tested species: mouse ;<br>Ashton JL, et al. American journal of physiology. Heart and circulatory physiology (2020) - IHC; tested species: rat; Banerjee A, Lee J, et al. eLife (2020) - ICC, IHC; tested species: mouse; Farassat N, et al. eLife (2019) - IHC; tested species: mouse; Liu C, et al. Cell (2018) - ICC, IHC; tested species: mouse;<br>González-Cabrera C, et al. The Journal of comparative neurology (2017) - IHC; tested species: mouse

Sc-48 Rabbit anti-FosB (Santa Cruz Biotechnology, Sc-398595, 1:100) has been discontinued and replaced by FosB (F-7) sc-398595 but its validity has been confirmed in a number of studies (e.g. PMID: # 24140894 González-Aparicio, R. et al. 2014. Neurobiology of disease; PMID: # 24259563 Lobo, MK. et al. 2013. J. Neurosci; PMID: # 23769604 Suárez LM, et al. 2013. Biol Psychiatry; PMID: # 29968767 Fieblinger T et al. 2018. Scientific Report.

Animals and other organisms

Policy information about studies involving animals; ARRIVE guidelines recommended for reporting animal research

Laboratory animals	As described in the "Online Methods" Section, L7-ChR2;WT mice were used for in vivo experiments, L7-ChR2;Drd2-GFP mice were used for ex vivo experiments. Animals were housed 1-3 per cage on a standard 12-hour light/dark cycle with ad libitum access to water and food and with a constant humidity of 40% and temperature of 22°C. All behavioral manipulations took place during the light phase. All experiments were performed on mice aged 6-9 weeks, of either sex (35-45g), from the Institut de Biologie de l'Ecole Normale Supérieure, Paris, France and in accordance with the recommendations contained in the European Community Council Directives
Wild animals	No wild animals were used in this study.
Field-collected samples	No field-collected samples were used in this study.
Ethics oversight	All experiments were performed in accordance with the recommendation containing in the European Community Council Directives and with proper authorizations (Ethics approval: APAFIS #29793-202102121752192 v3).

Note that full information on the approval of the study protocol must also be provided in the manuscript.
